# Cross-talk between transcriptome, phytohormone and HD-ZIP gene family analysis illuminates the molecular mechanism underlying fruitlet abscission in sweet cherry (*Prunus avium* L)

**DOI:** 10.1186/s12870-021-02940-8

**Published:** 2021-04-10

**Authors:** Zhilang Qiu, Zhuang Wen, Qiandong Hou, Guang Qiao, Kun Yang, Yi Hong, Xiaopeng Wen

**Affiliations:** Key laboratory of Plant Resource Conservation and Germplasm Innovation in Mountainous Region (Ministry of Education), Collaborative Innovation Center for Mountain Ecology & Agro-Bioengineering (CICMEAB), Institute of Agro-bioengineering/ College of Life Sciences, Guizhou University, Guizhou Province 550025 Guiyang, China

**Keywords:** Sweet cherry, Abscission, Phytohormone, Transcriptome, HD-ZIP

## Abstract

**Background:**

The shedding of premature sweet cherry (*Prunus avium* L) fruitlet has significantly impacted production, which in turn has a consequential effect on economic benefits.

**Result:**

To better understand the molecular mechanism of sweet cherry fruitlet abscission, pollen viability and structure had been observed from the pollination trees. Subsequently, the morphological characters of the shedding fruitlet, the plant hormone titers of dropping carpopodium, the transcriptome of the abscising carpopodium, as well as the HD-ZIP gene family were investigated. These findings showed that the pollens giving rise to heavy fruitlet abscission were malformed in structure, and their viability was lower than the average level. The abscising fruitlet and carpopodium were characterized in red color, and embryos of abscising fruitlet were aborted, which was highly ascribed to the low pollen viability and malformation. Transcriptome analysis showed 6462 were significantly differentially expressed, of which 2456 genes were up-regulated and 4006 down-regulated in the abscising carpopodium. Among these genes, the auxin biosynthesis and signal transduction genes (*α-Trp*, *AUX1*), were down-regulated, while the 1-aminocyclopropane-1-carboxylate oxidase gene (*ACO*) affected in ethylene biosynthesis, was up-regulated in abscising carpopodium. About genes related to cell wall remodeling (*CEL*, *PAL*, *PG EXP*, *XTH*), were up-regulated in carpopodium abscission, which reflecting the key roles in regulating the abscission process. The results of transcriptome analysis considerably conformed with those of proteome analysis as documented previously. In comparison with those of the retention fruitlet, the auxin contents in abscising carpopodium were significantly low, which presumably increased the ethylene sensitivity of the abscission zone, conversely, the abscisic acid (ABA) accumulation was considerably higher in abscising carpopodium. Furthermore, the ratio of (TZ + IAA + GA3) / ABA also obviously lower in abscising carpopodium. Besides, the HD-ZIP gene family analysis showed that *PavHB16* and *PavHB18* were up-regulated in abscising organs.

**Conclusion:**

Our findings combine morphology, cytology and transcriptional regulation to reveal the molecular mechanism of sweet cherry fruitlet abscission. It provides a new perspective for further study of plant organ shedding.

**Supplementary Information:**

The online version contains supplementary material available at 10.1186/s12870-021-02940-8.

## Background

Abscission is a highly programmed mechanism in which plants remove senescent, injured, infected, or dispensable organs, such as leaves, flowers, petals, sepals, and fruits [1]. The process involves cell separation which occurs in specialized cells in the petiole and pedicel, which is known as the abscission zone (AZ) [2]. The AZ consists of several layers of small cells, which are distinct from the surrounding cells, as well as these cells originally developed for the organ separation [3]. Earlier studies have shown that a few MADS-box genes have been related to the differentiation of AZ, including *JOINTLESS*, *MACROCAYLYX*, and *SVP* [4, 5]. After the AZ differentiation was complete, the AZ cells are predisposed to respond to shedding signals [2]. Under field conditions, the AZ firmly attaches the organ to the plant body [6]. The abscission process will be activated once AZ receives the abscission signals, such as growth, environmental stresses, senescence, and irregular fertilization [7].

Advancement in physiological, genetic, molecular, and biochemical approaches has significantly enhanced the understanding of abscission over the last few decades [8, 9]. In particular, in-depth research of model plants such as *Arabidopsis* and tomato makes it easier to understand the mechanism of plant organ shedding [10, 11]. Numerous studies have shown that plant hormones play a vital role in the shedding of plant organs [12, 13]. Phytohormones that occur in the plant are in equilibrium in normal conditions. However, some internal or external causes have disrupted the equilibrium, such as drought, starvation of carbohydrates, pests, diseases, and abnormal fertilization, and this occurrence has become a signal for plant organ shedding [7]. Among these phytohormones, ethylene (ETH) and abscisic acid (ABA) play a positive role in shedding [14]. In contrast, auxin (IAA), cytokinin (CTK) gibberellin (GA), and Polyamines (PAs) play a negative role in the abscission process [7]. Usually, the balance between ethylene and auxin plays a crucial role in the shedding process [1]. Besides, the lack of carbohydrates can also contribute to the abscission of plant organs [7, 15]. Consequently, genes that synthesize these phytohormones and signal transduction pathways also play a key role in the plant organ abscission mechanism [16, 17].

The plant cell wall consists of cellulose, hemicellulose, pectin, lignin, and structural proteins [18]. Upon induction, these cells secrete cell wall modifying and hydrolyzing enzymes, which loosen the cell wall and degrade the middle lamella between adjacent cells [2]. Studies have shown that some cell wall hydrolases and helper proteins can damage the structure of plant cell walls and eventually lead to the shedding of plant organs [19–21]. These hydrolases include cellulases (CELs), polygalacturonases (PGs), pectin methylesterases (PEMs), and pectate lyases (PLs). Moreover, some studies have shown that hydrolysis seems essential for cell expansion coordinated by other enzymes like expansins (EXPs), as well as xyloglucan endotransglucosylase/hydrolases (XTHs) [21]. After degradation of the cell wall and abscission of the plant organs, the formation of a protective layer may mitigate water loss and create a physical barrier against opportunistic pathogen attacks [22]. To support anatomical findings for the formation of the protective layer in the AZ, it has been shown that stress-related peroxidase activity within AZ is increased, which is assumed to play a role in the lignification process of AZ [23].

Based on years of previous research, a regulatory model for plant organ shedding has been established [24]. Abscission is an organized, regulated developmental process consisting of four discernible steps: (1) differentiation of the AZ; (2) acquisition of competence to respond to abscission signals, (3) activation of plant organs abscission, and (4) post-abscission differentiation of a protective layer. In the first step, *JOINTLESS* interacts with the MADS-box gene *MACROCALYX* to regulate the development of the abscission zone in the flower pedicel [5]. The *LeACO* will enhance the ethylene biosynthesis in the next step, which can be a signal to stimulate the abscission region [16, 25]. In the third step, when plant organs sense abscission signals, some genes are expressed specifically in the AZ, and the abscission of organs was activated. For example, the *AtDOF4.7* can act as a positive regulator by directly binding to the promoter of *ADPG2* [10]. Another, the *LcHB2* can act as a positive regulator of fruitlet shedding through directly activating *LcCEL2* and *LcCEL8* in litchi, and cellulase activities are increased. As a consequence, the cellulose content is reduced and the fruitlet shedding is ultimately induced by the deterioration of the cell wall and cell separation in the abscission zone [26]. In the last step, the differentiation of a protective layer were involves programmed cell death (PCD), which involves the induction of nucleases and reactive oxygen species (ROS) [27].

Sweet cherry (*Prunus avium* L.) is one of the most popular fruits containing various sugar (glucose, fructose, sucrose, and sorbitol), dietary fiber, and melatonin [28], it also is rich in melatonin and dietary fiber, simple sugars, sweet cherry also contains various vitamins, minerals primarily potassium, phenolic compounds (flavonoids and anthocyanins). Moreover, eating sweet cherries can reduce the risk of cancer and joint pain, as well as prevent neurodegenerative diseases [29]. Owing to the productive nutritional value of sweet cherry, it is extensively cultivated in countries such as China, America, and Japan, Chile. However, fruitlet shedding dramatically reduces yield and economic efficiency. Therefore, efforts on revealing the molecular mechanism of sweet cherry fruitlet shedding can facilitate to attempt to develop high-yield cultivars.

In the present study, the pollen vitality, the transcriptome, phytohormone content, and the HD-ZIP gene family in sweet cherry were be analyzed. (1) This study found that the pollen vigor of the ‘Brooks’ was lower, and the structure of the pollen electron microscope was deformed; (2) The transduction of plant hormone signals and genes linked to cell wall modifications is significantly up-regulated in abscising carpopodium; (3) the *PavHB16,* cell wall remodeling protein, and plant hormone biosynthesis and signal transduction were regulated significantly between transcriptome and proteome; (4) the *PavHB16* and *PavHB18* were up-regulated in abscising organs (flower, fruitlet, carpopodium, leaf, and petiole). These results contribute to our understanding of the transcriptional regulatory mechanisms of the sweet cherry fruitlet abscission.

## Results

### Pollen vitality of the main pollination tree

**Fig. 1:**
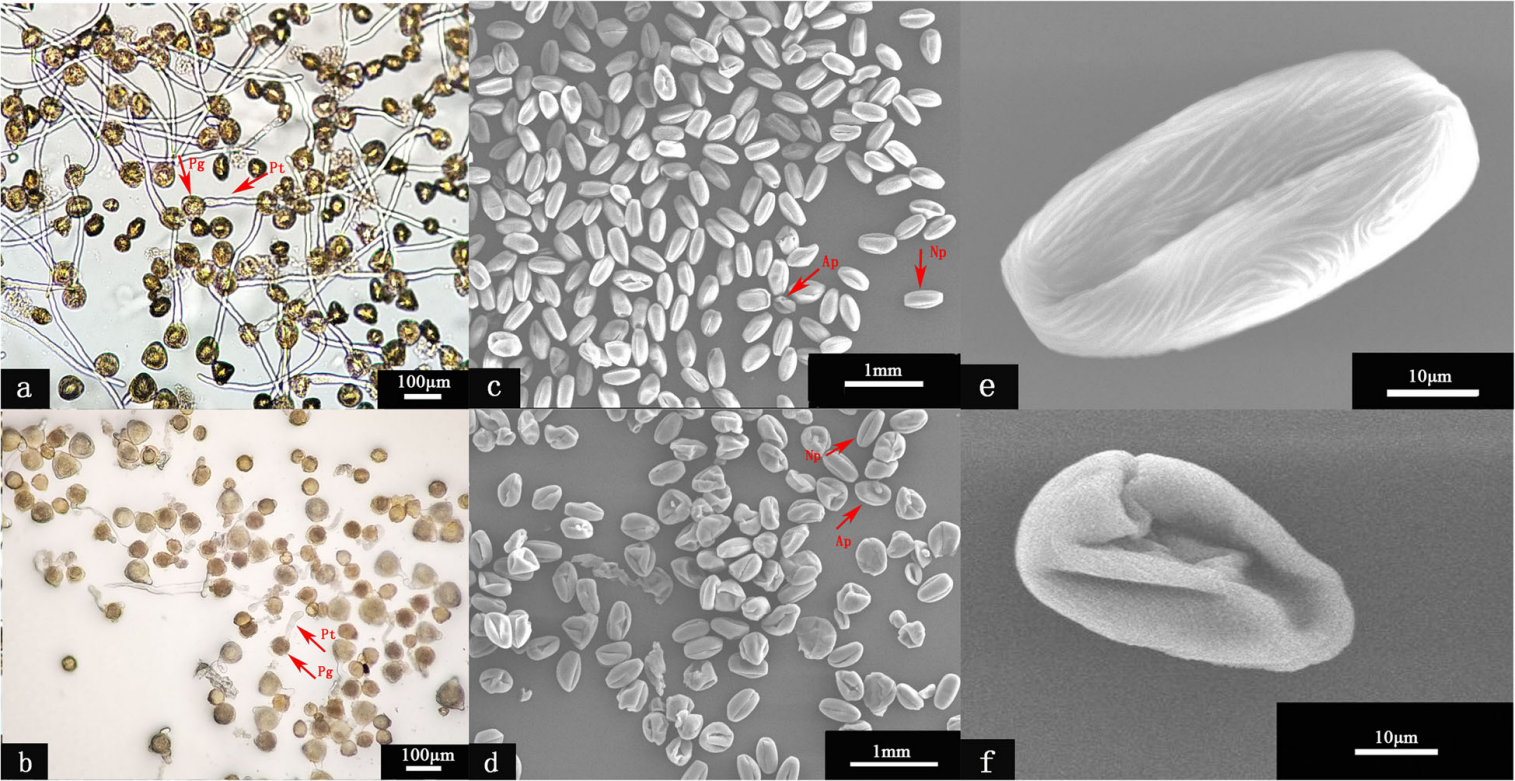
The pollen vigor under the optical microscope and structure under the scanning electron microscope. (**a**) Pollen tube germination of Qianying No. 1; (**b**) Pollen tube germination of Brooks; (**c**) Electron microscopic structure of pollen of Qianying No. 1; (**d**) Electron microscopic structure of pollen of Brooks; (**e**) Electron microscopic structure of normal pollen; (**f**) Electron microscopic structure of deformity pollen. Pg: pollen grain; Pt: pollen tube; Ap: abortion pollen; Np: normal pollen

The results showed that the pollen germination rate of ‘Qianying No. 1’ accounted for 66.3% (Fig. 1 a), while the pollen of ‘Brooks’ only accounted for 13.5% (Fig. 1 b). According to the pollen’s morphological characteristics, it can be divided into normal pollen (Fig. 1 e) and deformity pollen (Fig. 1 f). According to the statistics, the deformity rate accounted for 13.33% in the ‘Qianying No. 1’ (Fig. 1 c), while that in the ‘Brooks’ accounted for 88.81% (Fig. 1 d) (Additional file 1: Table S1). These results suggest that pollen activity may have a great correlation with pollen morphology. In other words, the higher the pollen vigor, the lower the pollen deformity rate. And this lower pollen vigor of ‘Brooks’ pollination trees will lead to embryo abortion, which is a rationale for sweet cherry fruitlet abscission.

### Morphology and anatomical structure of abscission fruitlet

**Fig. 2:**
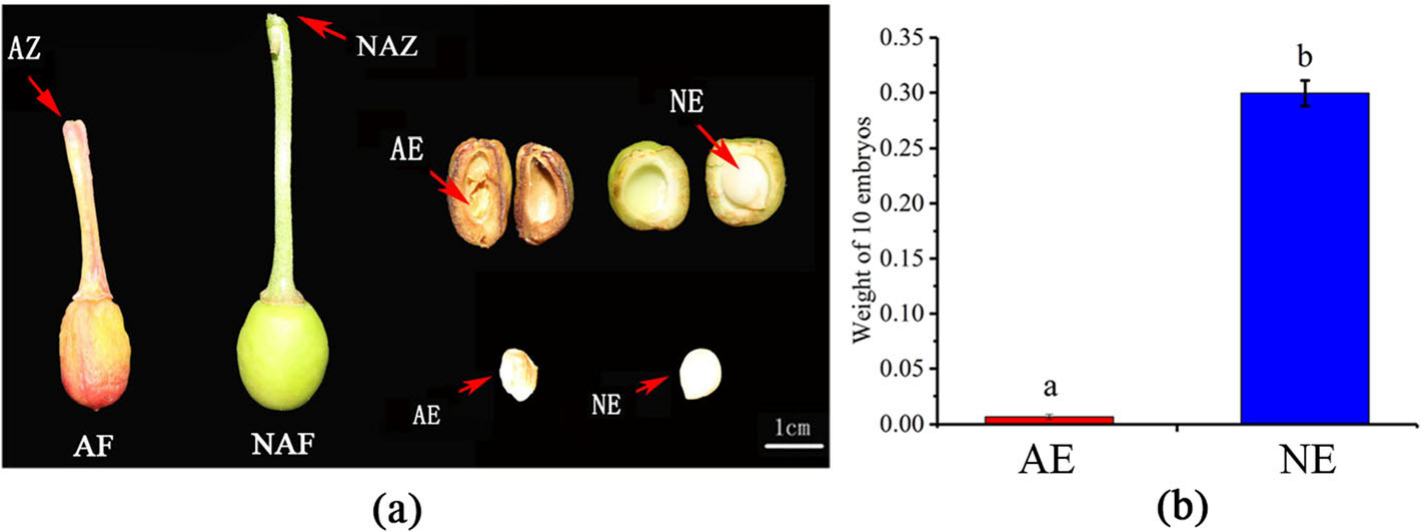
The morphological characteristics and the weight of ten embryos of abscising sweet cherry fruitlet. **a** AZ: abscising carpopodium abscission zone; NAZ: non-abscising carpopodium abscission zone; AF: abscising fruitlet; NAF: non-abscising fruitlet; AE: abscising fruitlet embryo; NE: non-abscising embryo. **b** Different letters indicate significant pairwise differences according to Duncan’s test (*P* < 0.05)

The morphological structure of the shedding fruitlet and carpopodiums was red and the embryos dried out based on fruitlet morphological character observations. In contrast, the retention of fruitlet and carpopodiums were green and the embryos were full (Fig. 2a). Additionally, the weight of ten abscising embryos (0.0068 g) was conspicuously lower than that of non-abscising embryos (0.3000 g) (Fig. 2b). These results suggest that the embryo of the shedding fruitlet has aborted, which can trigger hormone imbalance and lead to the fruitlet being prematurely shedding. Such findings indicate that there be an excellent correlation between the shedding of sweet cherry fruitlet and embryo abortion.

### Endogenous hormone analysis between the abscising and non-abscising carpopodium

**Fig. 3:**
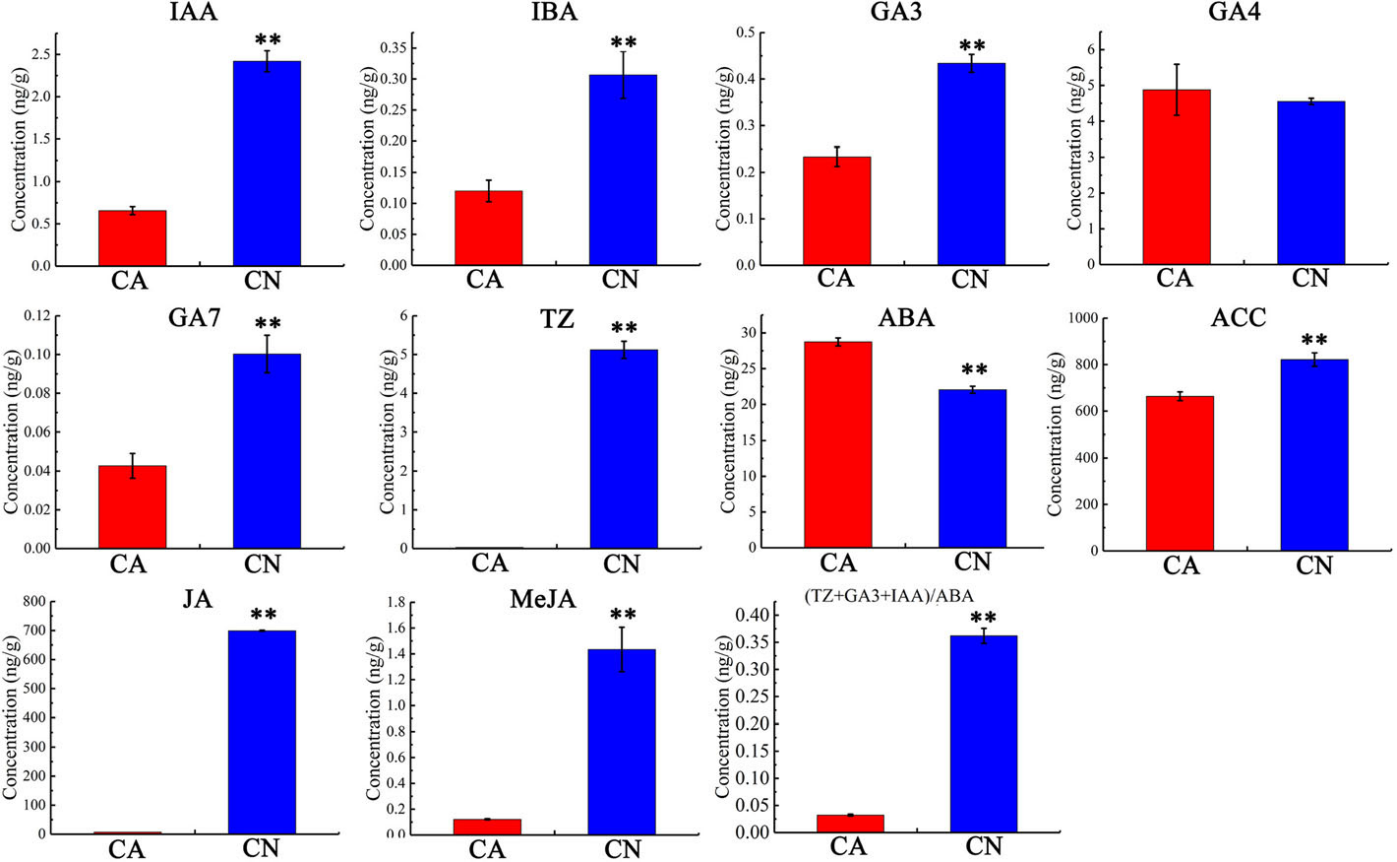
Endogenous hormone analysis between the abscising carpopodium (CA) and non-abscising carpopodium (CN). Auxin: IAA (indole-3-acetic acid); IBA (indole-3-butyric acid); Gibberellin: GA3, GA4, GA7; Cytokinin (CTK): TZ (Trans-zeatin); Abscisic acid: ABA; 1-aminocyclopropane-1-carboxylate: ACC; Jasmonic acid: JA; Methyl jasmonate: MeJA. ** indicate significant differences in comparison with values at CA at *P* < 0.01 (t-test)

To further study the hormonal regulation of physiological fruitlet abscission in sweet cherry, endogenous levels of Auxin: Indole-3-Acetic Acid (IAA) and Indole-3-butyric acid (IBA), gibberellins (GAs): GA3, GA4, and GA7, cytokinins: trans-zeatin (TZ), abscisic acid (ABA), 1-aminocyclopropane-1-carboxylate (ACC), jasmonic acid (JA), and Methyl jasmonic acid (MeJA) were analyzed between the abscising carpopodium and non-abscising ones in the fruitlet development stage, which precede the physiological abscission. The distributions of auxins (IAA, IBA) in abscising carpopodium were stated to be lower compared to non-abscising carpopodium. Out of the three GAs analyzed, GA4 was detected at similar levels in both abscising carpopodium and non-abscising carpopodium, while the GA3 and GA7 levels significantly decreased in abscising carpopodium. Cytokinins (TZ) levels were found to reduce notably in abscising carpopodium. However, the abscisic acid level was markedly increased in abscising carpopodium. Incredibly, the levels of 1-aminocyclopropane-1-carboxylatewere (ACC), jasmonic acid (JA), and Methyl jasmonic acid (MeJA) decreased prominently in abscising carpopodium. To observe the balance of plant hormones comprehensively, the ratio of (TZ + IAA + GA3) / ABA was calculated, and the results showed that the ratio in abscising carpopodium was significantly lower than that of non-abscising carpopodium (Fig. 3, Additional file 2: Table S1). These findings indicate that the reduced auxin can increase the sensitivity of ethylene abscission zone reaction, and the increased content of ABA will also expedite the abscission of sweet cherry fruitlet. However, in the abscising carpopodium, the content of GA3 and CTK related to inhibition of sweet cherry fruitlet abscission is lower. The speculate for sweet cherry fruitlet shedding maybe all these hormonal changes, although the main cause may be the auxin decline.

### Transcriptome profiling and identifying differentially expressed genes (DEGs)

Transcriptomes of abscising carpopodium and non-abscising carpopodium were analyzed using RNA-seq to obtain detailed and effective transcriptome data for sweet cherry fruitlet abscission. Before RNA-seq analysis, six cDNA libraries were constructed and generated paired-end sequence reads using the Illumina Hiseq 4000 platform. The raw data have been deposited in NCBI Sequence Read Archive (SRA) through Gene Expression Omnibus (GEO) (access number: PRJNA636209). A sum of 3.07 billion raw reads was generated and each sample provided an average production of 51.33 million. A mean of 50.54 million clean reads was obtained from each library with an adequate read ratio of 98.48% after removing adaptor sequences, N-containing reads, and low-quality. (Additional file 3: Table S3). The reference genome exactly matched the average mapping ratio of 89.87% to roughly 45.41 million clean reads across each library. The Pearson correlation coefficient with all gene expression levels between every three samples was determined to investigate the gene expression correlation between samples. (Additional file 4: Figure S1).

**Fig. 4:**
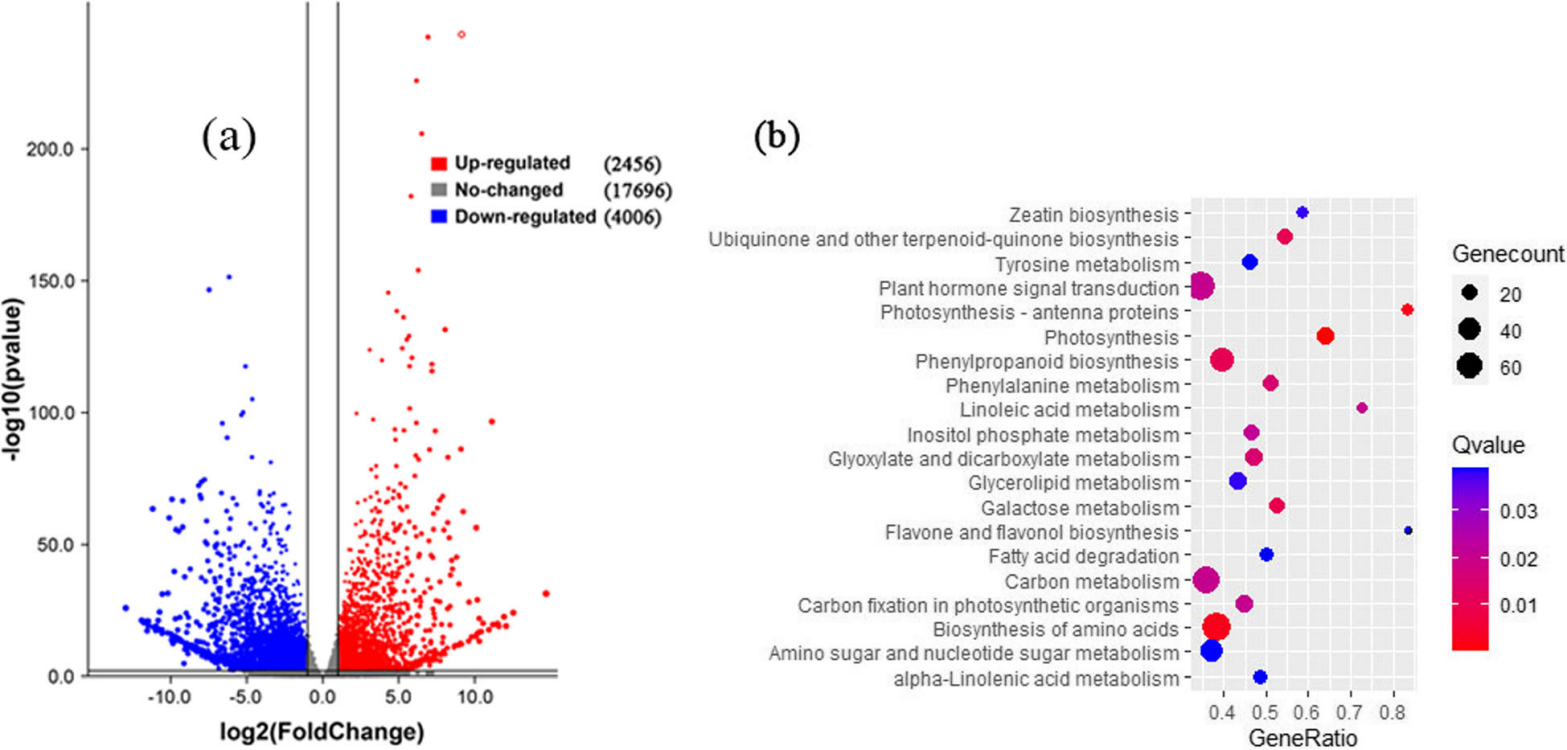
The Volcano diagram of differentially expressed genes between CA and CN **(a)** and DEGs involving in KEGG pathway enrichment analysis in comparison with CA and CN **(b)**. In **(a)**, The x-axis represents the multiple differential expressions; the y axis represents significance. In **(b)**, The x-axis represents the GeneRatio, GeneRatio = Term Candidate Gene Number/Term Gene Number. The Y-axis represents KEGG Pathway. The size of the bubble is proportional to the number of genes in the KEGG Pathway. And the color represents the Q-value of enrichment. The deeper the color, the smaller the Q-value

A total of 43,673 genes were mapped from all the samples. Abscising and non-abscising carpopodium gene expression levels were analyzed, and 6462 differentially expressed genes (DEGs) were recognized. (Additional file 5: Table S4). Among such DEGs, the abscising carpopodium had 2456 DEGs up-regulated and 4006 DEGs down-regulated. (Fig. 4 a). The number of down-regulated DEGs was higher than that of up-regulated ones; It is worth pointing out that some genes related to plant cell wall remodeling have been significantly up-regulated. This may be the most direct factor regulating sweet cherry fruitlet shedding.

### Enrichment analysis of DEGs during carpopodium abscission

The KEGG pathways analysis was performed; in the light of these enrichment results, the top 20 pathways among abscising with non-abscising carpopodium were shown in Fig. 4 b and Additional file 6: Table S5. In these pathways, the plant hormone signal transduction and galactose metabolism were involved, which may regulate sweet cherry fruitlet abscission. Besides, the auxin and ethylene biosynthesis relate pathways were also enriched (Additional file 6: Table S5). Additionally, some pathways associated with cell wall modification were also augmented. These results indicated that phytohormone biosynthesis and signal transduction, and cell wall modification play crucial roles in fruitlet abscission regulation. These findings coincide with previous studies on the pathway [30, 31]. Moreover, there were other pathways including biosynthesis of amino acids, carbon metabolism, and phenylpropanoid biosynthesis was found to be enriched.

**Fig. 5:**
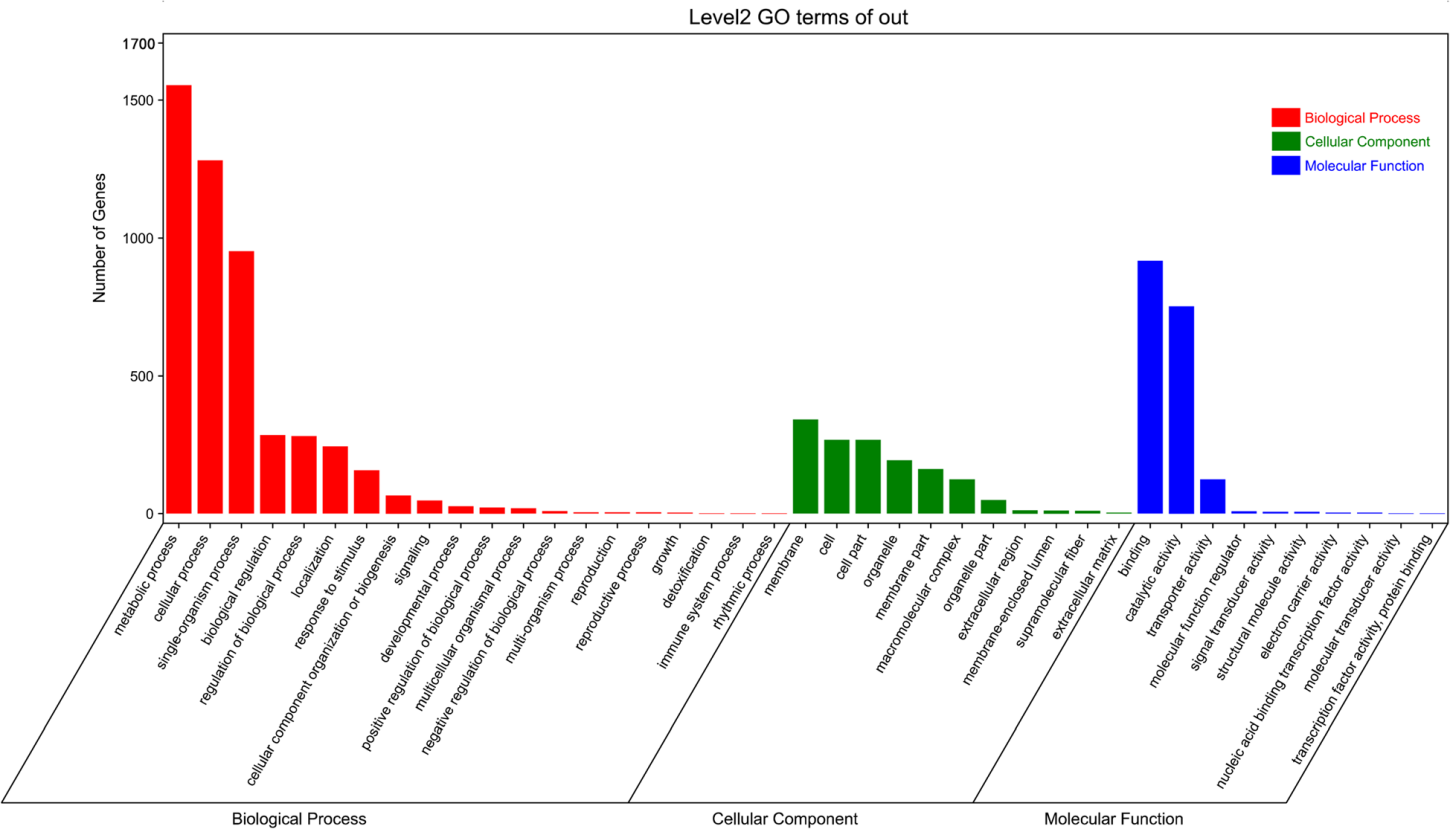
GO classification of DEGs. The X-axis represents the functional classification, and the Y-axis represents the number of genes annotated into the GO terms

The Gene Ontology (GO) classification results indicated that 2596 DEGs between the abscising and non-abscising carpopodium were graded into three varieties: biological process (BP), cellular component (CC), and molecular function (MF) (Additional file 7: Table S6). In the BP category, the metabolic process and the cellular process were the most plentiful terms. For the CC variety, membrane, cell, and cell parts were the major terms. The top three terms of MF were binding, catalytic activity, and transporter activity (Fig. 5).

### Plant hormone biosynthesis and signal transduction

The KEGG enrichment analyses showed “plant hormone signal transduction” was a significant pathway. In the auxin signal transduction pathway, the significantly down-regulated genes include auxin influx carrier (AUX1), Auxin/Indole-3-Acetic Acid (AUX/IAA), auxin response factor (ARF), and small auxin up RNA (SAUR). In the cytokinin signal transduction pathway, the cytokinin receptor CRE1 was down-regulated. However, in the ABA signal transduction pathway, the PP2C and SnRK2 were up-regulated in abscising carpopodium. Simultaneously, in the ethylene signal transduction pathway, the ethylene insensitive 3 (EIN3) and ethylene response factor (ERF) were up-regulated which may improve the fruitlet abscission. Also, some essential genes are differentially expressed in the plant hormone synthesis pathways. These genes include the tryptophan synthase alpha chain (α-Trp), which is related to auxin biosynthesis. Additionally, the 1-aminocyclopropane-1-carboxylate synthase and 1-aminocyclopropane-1-carboxylate oxidase (ACO) were up-regulated in abscising carpopodium.

### Cell wall remodeling related genes

Cell wall remolding was one of the methods employed by cells to regulated abscission. Our RNA-Seq analyses of abscising carpopodium and non-abscising ones showed bidirectional changes in the expression of the cell wall remodeling genes. This phenomenon may be associated not only with the ongoing process of abscission but also with the progressive development of the organs that are not dropped (Additional file 8: Table S7). Among these cell wall remodeling-related DEGs, there were 4 cellulases (CELs), 7 polygalacturonases (PGs), 5 pectinases (PEs), 7 peroxidases (PODs), 2 beta-galactosidase (BGALs), 5 expansins (EXPs), and 5 xyloglucan endotransglucosylase/hydrolase (XTHs) was up-regulated, which may be regulated the cell wall remodeling. These results indicate that cell wall remodeling-related genes play a vital role during fruitlet abscission. Regardless of the differential expression of these cell wall remodeling enzyme genes, the cell wall was remodeled, leading to the degradation of the cell wall or middle lamella, which leads to cell separation and fruitlet shedding.

### Transcription factor

**Fig. 6:**
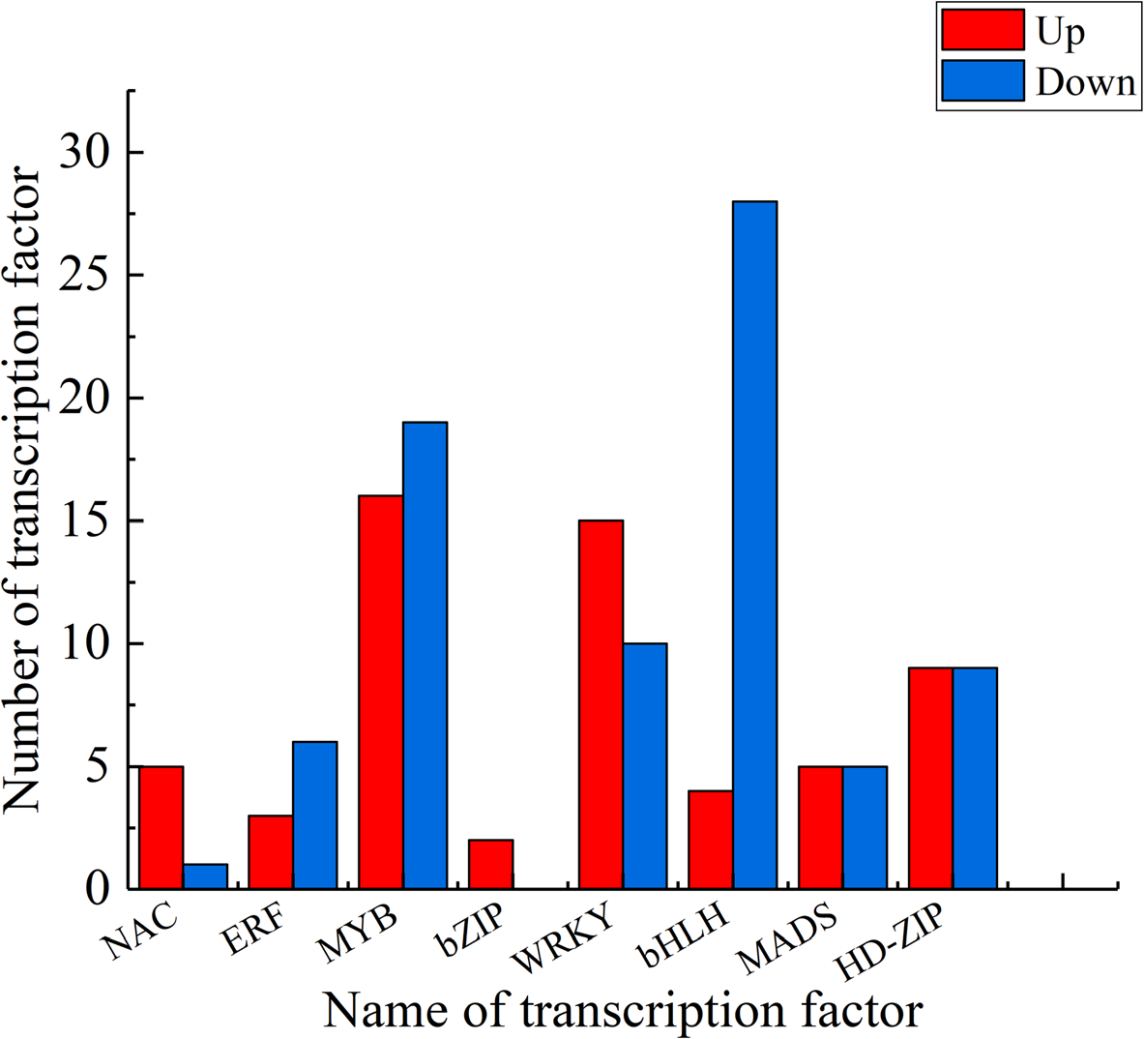
Eight major transcription factors are differentially expressed in abscising and non-abscising carpopodium. The X-axis represents the class of transcription factor, and the Y-axis represents the number of the transcription factor

Transcriptional regulation plays a pivotal role in the complex series of events leading to plant organ abscission. Therefore, transcription factors also enact an imperative role in the process. According to our data, there were 8 types of transcription factors that deserve attention, namely NAC, ERF, MYB, bZIP, WRKY, bHLH, MADS, HD-ZIP, which may regulate the shedding of sweet cherry fruitlet. Among these transcription factors, the most significant number of differential expressions was MYB, followed by WRKY and HD-ZIP (Fig. 6). It is noteworthy that the genes of these three gene families are likely to regulate the shedding of plant organ abscission. Moreover, by influencing plant hormone biosynthesis genes and cell wall modification associated with enzyme genes, the HD-ZIP family has been shown to induce shedding in litchi.

### Cross-talk between carpopodium transcriptome and proteome

**Fig. 7:**
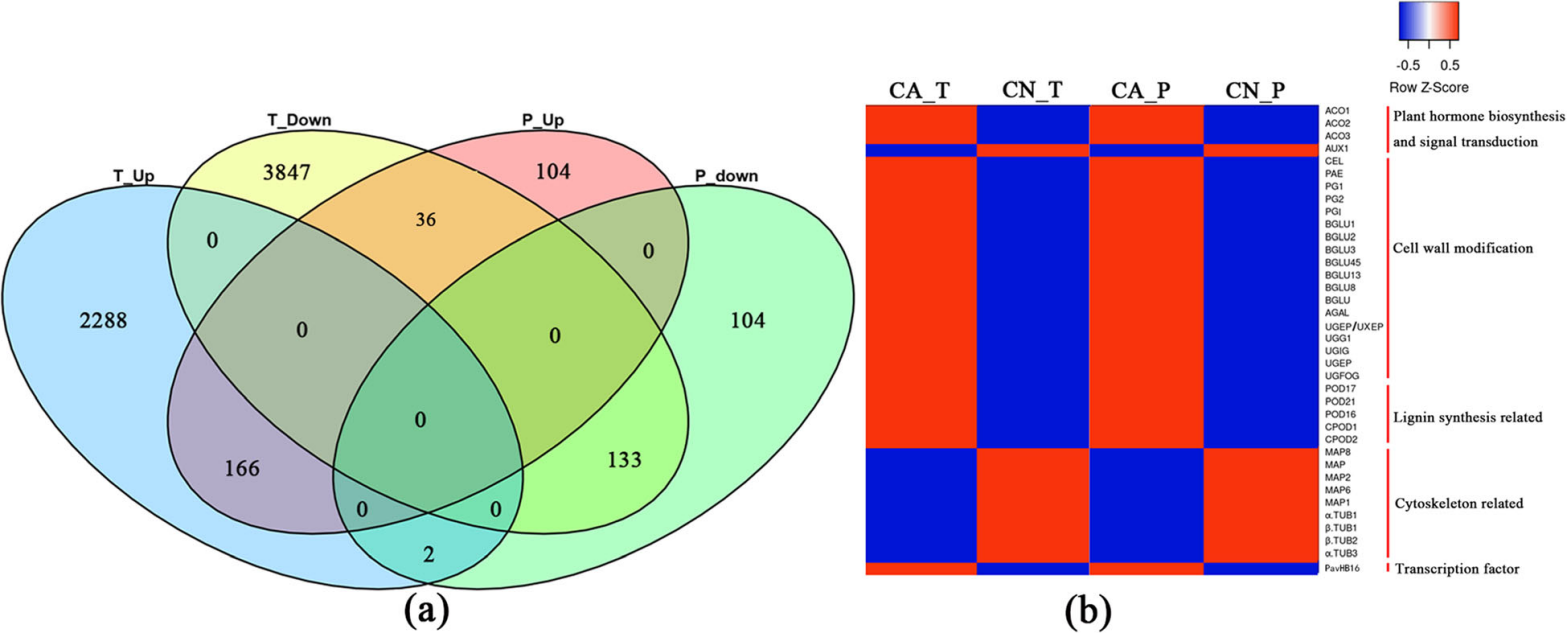
The Venn diagram between transcriptome and proteome **(a)** as well as expression analysis between abscising carpopodium and non-abscising carpopodium **(b)**. T_Up and T_Down represent the genes up-regulated and down-regulated at the transcriptional level of abscising carpopodium respectively; P_Up and P_Down represent the proteins up-regulated and down-regulated at the protein level of abscising carpopodium respectively; CA_T and CN_T represent the relative expression levels of the transcriptome in abscising and non-abscising carpopodium respectively; CA_P and CN_P represent the relative expression levels of the proteome in abscising and non-abscising carpopodium respectively

This research performed joint proteomics and transcriptomics assessment to more explicitly find out the primary genes of sweet cherry fruitlet shedding. This research performed joint proteomics and transcriptomics assessment to more explicitly find out the primary genes of sweet cherry fruitlet shedding. The differentially accumulated proteins (DAPs) with 1.5-fold and differentially expressed genes with 2 folds are being used for cooperative investigation. There were 337 genes/proteins common differential expression between the transcription level and the protein level. Among these genes, there were 166 genes frequent up-regulation and 133 genes common down-regulation (Fig. 7 a). It is evident that in the plant hormone biosynthesis and signal transduction pathways, three genes linked to ethylene biosynthesis, 1-aminocyclopropane-1-carboxylate oxidase (ACO), were identified to be up-regulated in the abscising carpopodium; while one auxin efflux carrier (AUX1) gene was down-regulated which might involve in auxin transport. More significantly, enzymes relevant to the plant cell walls remodeling, such as cellulose, pectin acetylesterase, and polygalacturonase have been up-regulated. Likewise, peroxidase associated with lignin biosynthesis in plant cell walls is also being up-regulated. However, some tubulins related to cell wall synthesis including tubulin alpha chain (α-TUB), tubulin beta chain (β-TUB), and microtubule-associated proteins (MAPs) showed a downward trend. Excitingly, a homeobox leucine zipper transcription factor (*PavHB16*) was up-regulated at both transcription and protein levels (Fig. 7 b).

### Verification of differential expression gene by quantitative real-time PCR

**Fig. 8:**
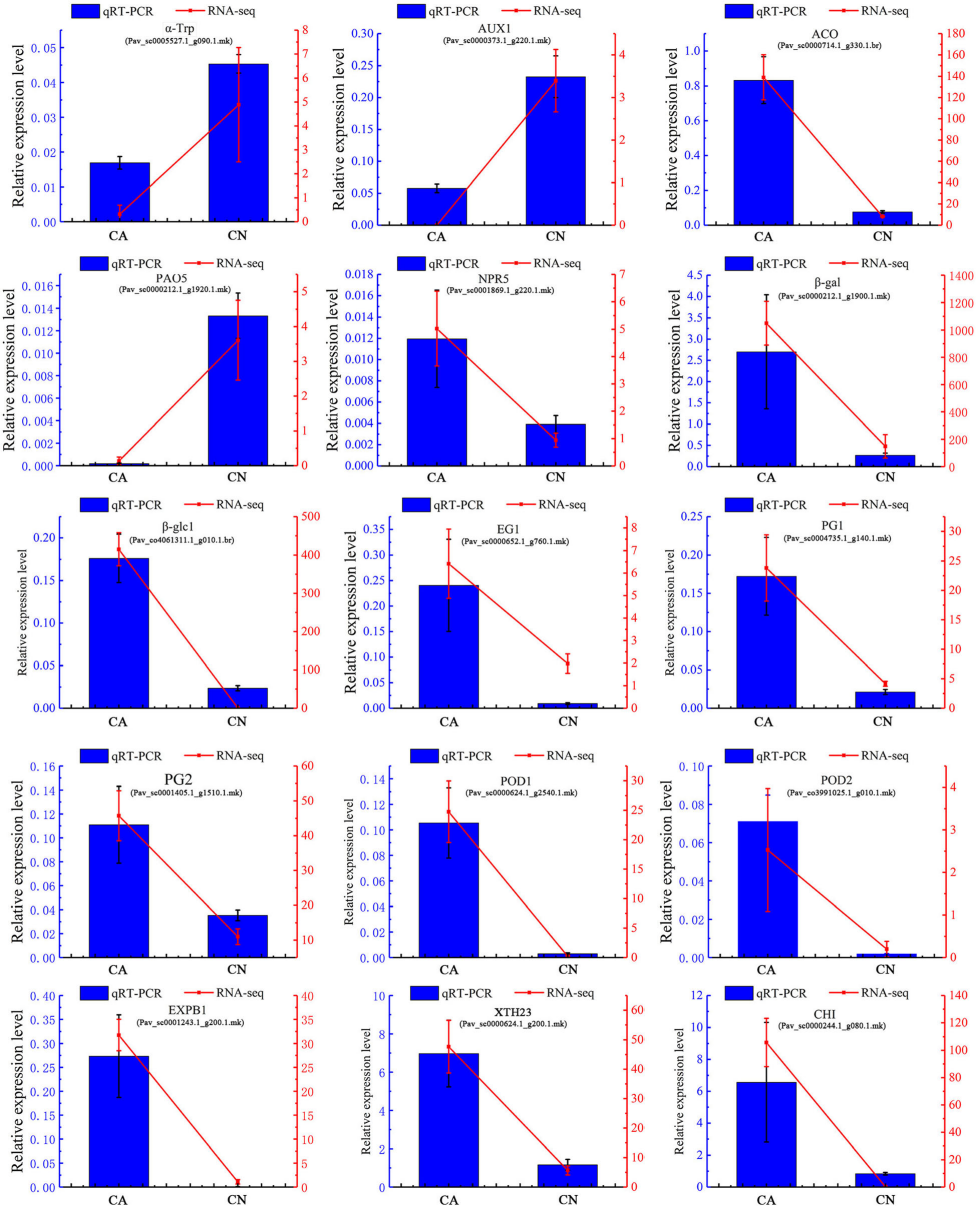
Relative expression levels of 15 DEGs in abscising carpopodium and non-abscising carpopodium. α-Trp: tryptophan synthase alpha chain; AUX1: auxin influx carrier; ACO: 1-Aminocyclopropane-1-Carboxylate Oxidase; PAO5: polyamine oxidase 5; NPR5: Non-expressor of PR5; β-gal: beta-galactosidase; β-glc1: beta-glucanase; EG1: endoglucanase; PG: polygalacturonase; POD: peroxidase; EXPB1: expansin-like B1; XTH23: xyloglucan endotransglucosylase/hydrolase 23; CHI: endochitinase

To confirm findings of gene expression obtained from transcriptome data, 15 DEGs concerned to plant organ abscission were chosen for qRT-PCR. These DEGs are mainly involved in phytohormone biosynthesis, plant hormone signal transduction, plant cell wall remodeling, which may regulate the abscission of sweet cherry fruitlet. As shown in, the 15 DEGs had very similar expression patterns based on the transcriptome data and qRT-PCR results, which indicates the trustworthiness of the transcriptomic analysis (Additional file 9: Table S8, Fig. 8).

### Identification and expression profiling of HD-ZIP gene family

**Fig. 9:**
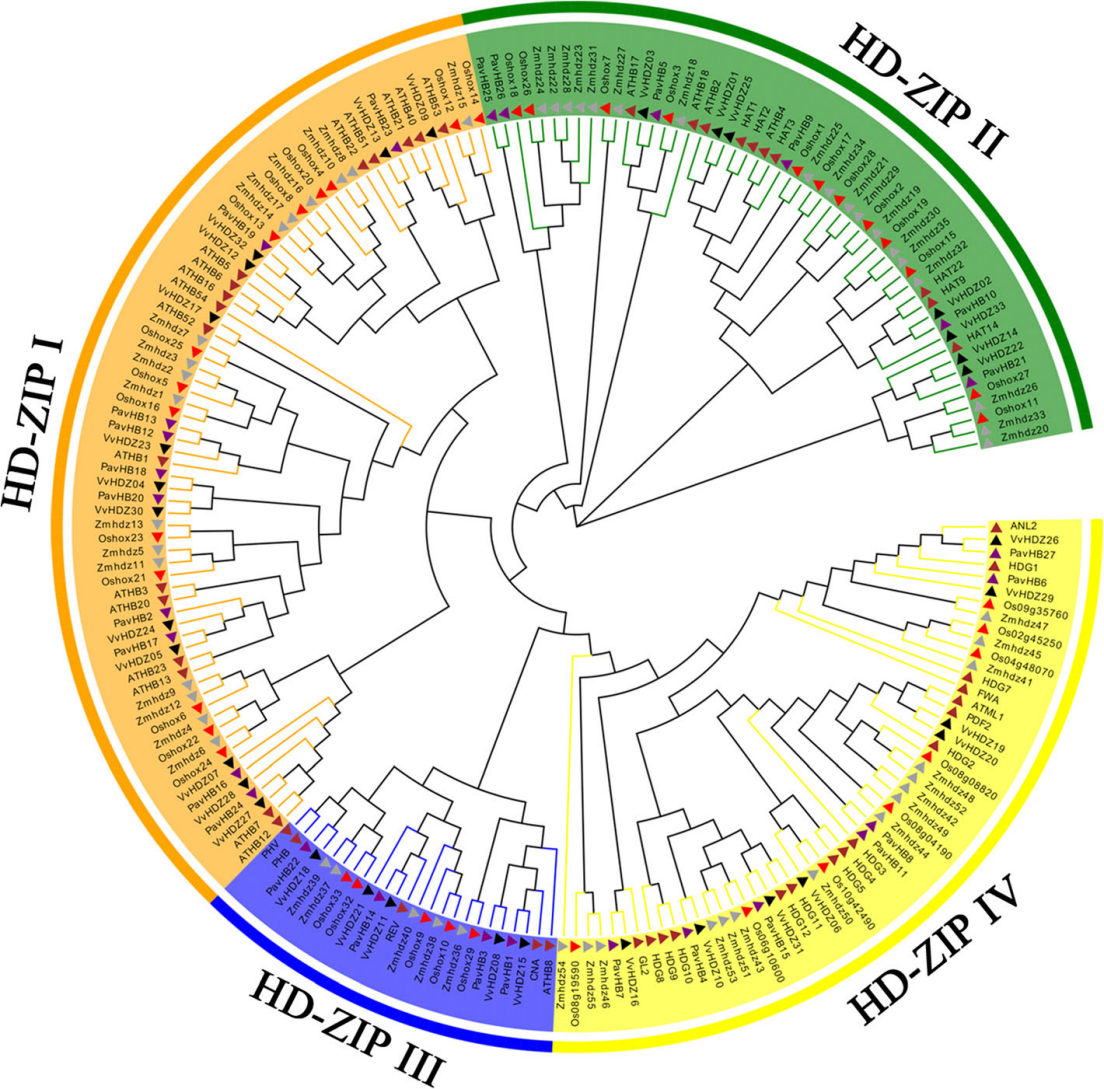
The phylogenetic tree of sweet cherry HD-ZIP genes (a). Members of the HD-ZIP genes from sweet cherry, Arabidopsis, maize, grapevine and rice are marked: purple, brown, grey, black and red triangles, respectively. The phylogenetic tree was generated by MEGA 7.0 using the Neighbor-Joining method, bootstrap test (1000 replicates)

**Fig. 10:**
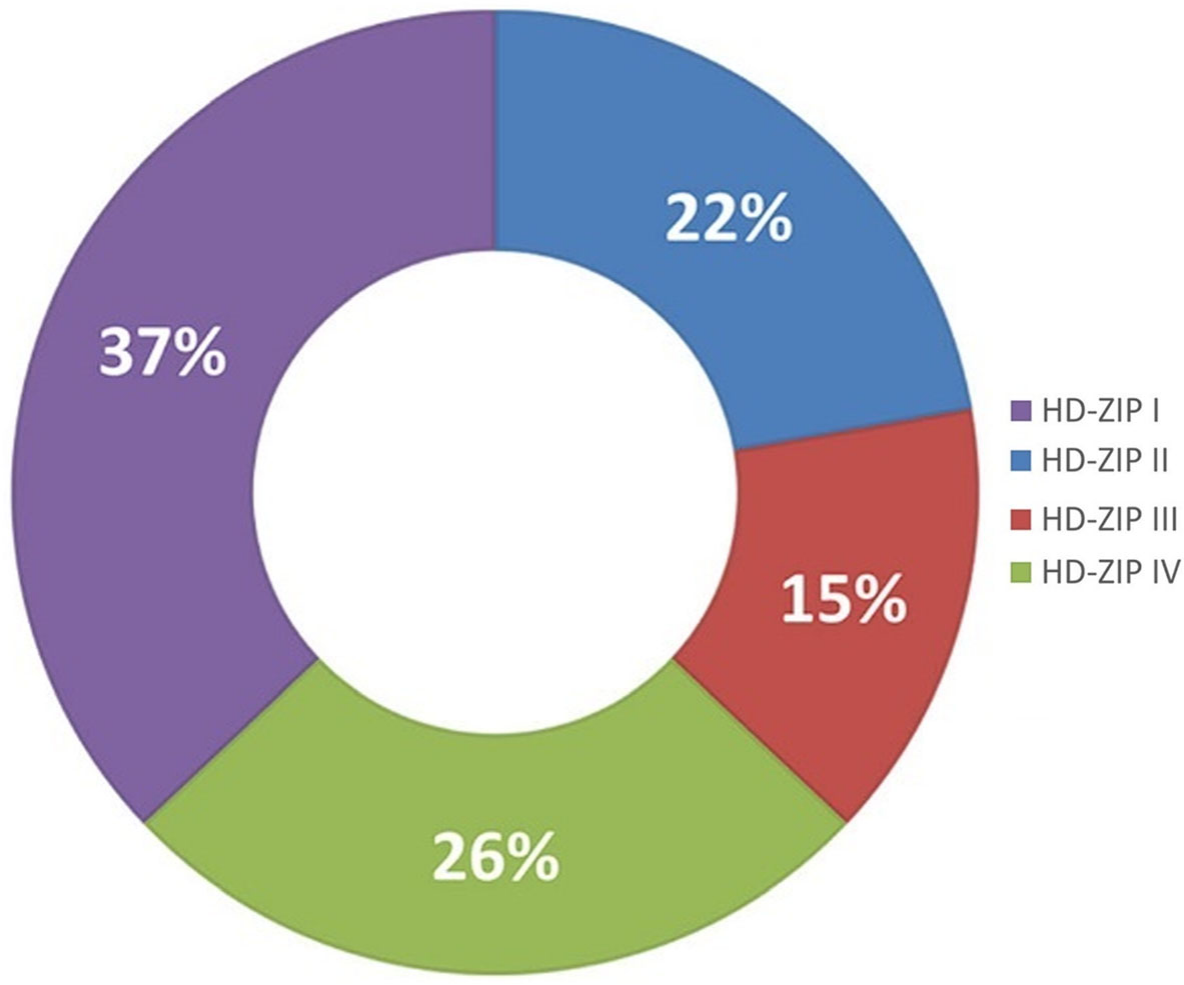
Proportions of various HD-ZIP classes in sweet cherry. The proportions of various HD-ZIP classes were performed by Micro software Excel 2016

**Fig. 11:**
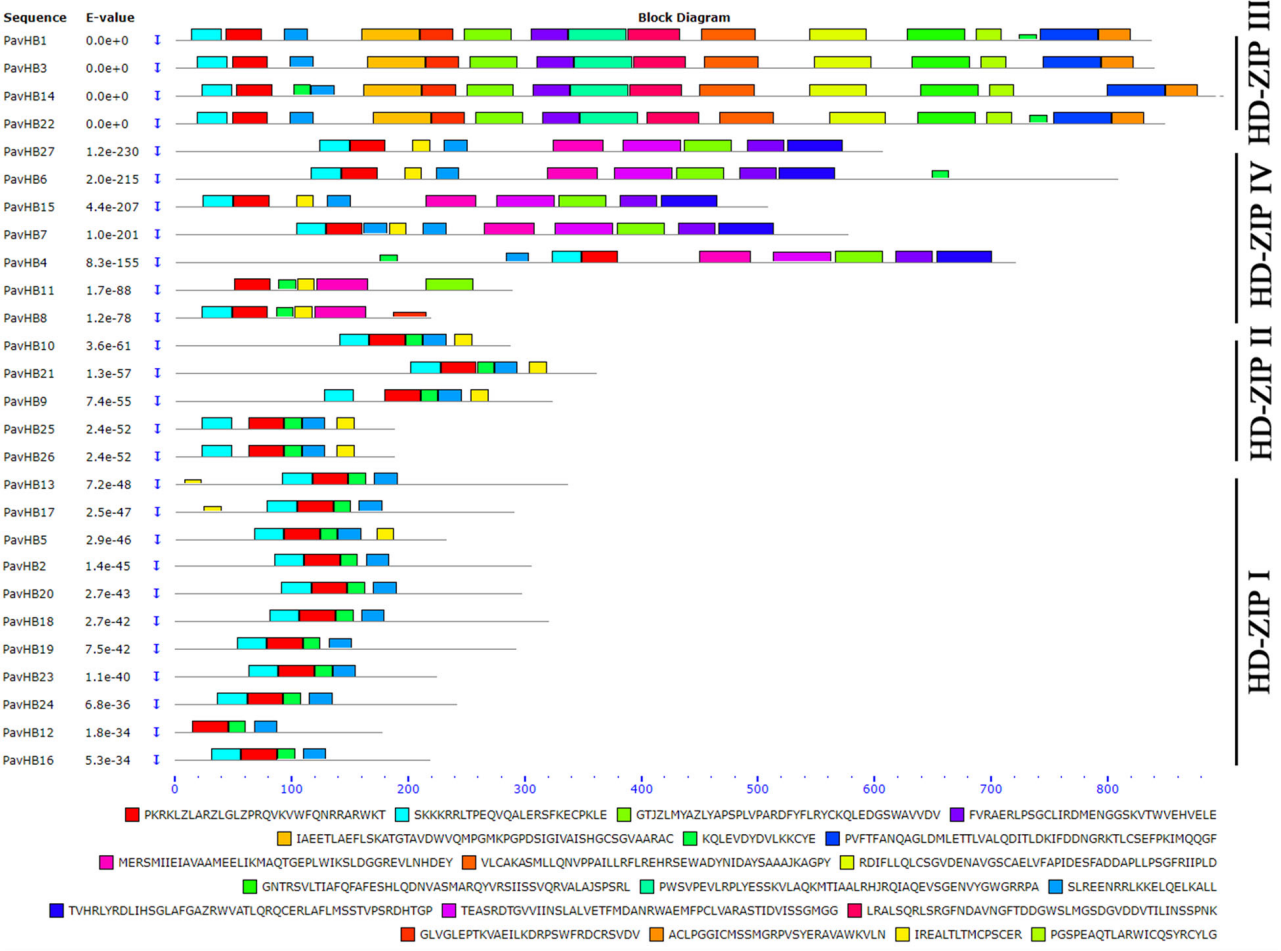
Common motifs of HD-ZIP family proteins in *Prunus avium* L

According to previous studies [26, 32], the HD-ZIP gene family plays an influential, role in the shedding of plant organs. To explore the relationship between HD-ZIP and sweet cherry fruitlet shedding, this study identified the HD-ZIP gene family of sweet cherry. The phylogenetic tree was constructed with HD-ZIP TFs in sweet cherry, Arabidopsis, maize, grapevine, and rice (Fig. 9). The proportion of each sub-family of HD-ZIP was calculated in the sweet cherry (Fig. 10). The result showed that the sweet cherry HD-Zip TFs were divided into four subgroups (I, II, III, and IV). In this sub-family, HD-Zip I (ten members), which accounted for 37%, was the largest class of PavHB TFs, followed by HD-ZIP IV and HD-ZIP II, with 26% (seven members) and 22% (six members), respectively; the smallest was HD-Zip III (four members), with just 15%. According to the gene structure analysis, HD-Zip III and HD-Zip IV had more motifs than the other two groups, and most proteins in HD-Zip III had 15 or 16 motifs. Additionally, the same subfamily with similar motifs (Fig. 11). Among these HD-ZIPs, it is noticeable that *PavHB16*, which was up-regulated in the transcriptome and the proteome, belongs to the HD-ZIP I subgroup.

**Fig. 12:**
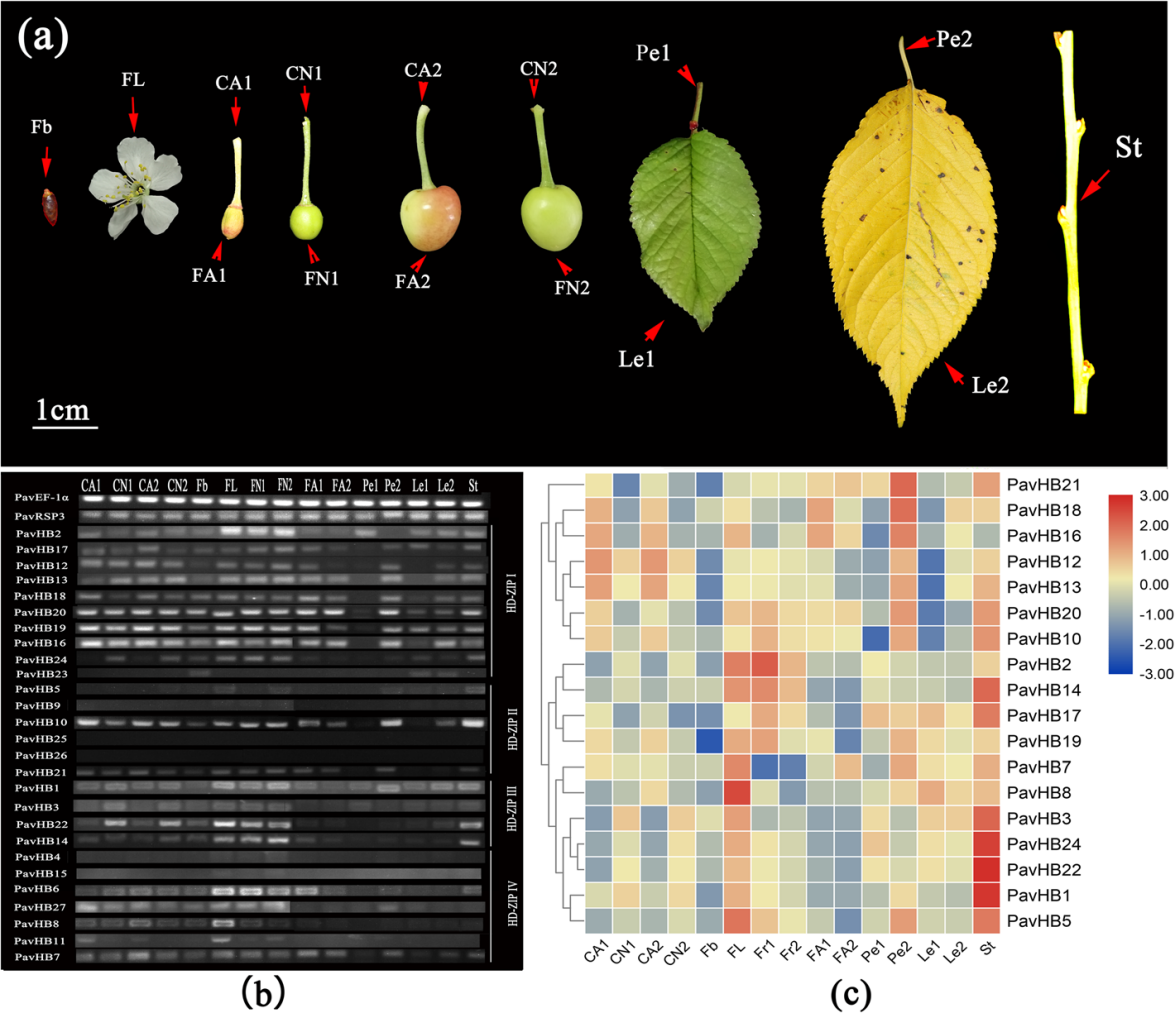
Gene expression levels in different tissue. **a** Different tissue of sweet cherry; **b** The semiquantitative of HD-ZIP gene family in different tissue (the original gel images were shown in Additional file 10: Figure S2); **c** The heatmap representing the expression patterns of HD-ZIP gene family*.* Fb: Flower bud; FL: Flower; CA1: Abscising carpopodium in the first stage; CN1: Non-abscission carpopodium in the first stage; FA1: Abscising fruitlet in the first stage; FN1: Non-abscising fruitlet in the first stage; CA2: Abscising carpopodium in the second stage; CN2: Non-abscission carpopodium in the second stage; FA2: Abscising fruitlet in the second stage; FN2: Non-abscising fruitlet in the second stage; Pe1: Young leaf petiole; Pe2: Old leaf petiole; Le1: Young leaf; Le2: Old leaf; St: Stem

**Fig. 13:**
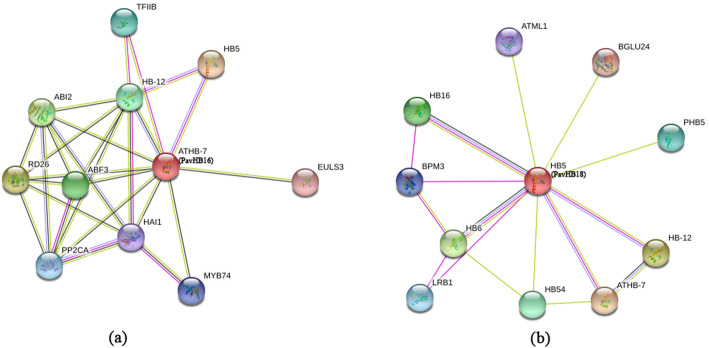
Interaction networks of *PavHB16* (**a**) and *PavHB18* (**b**) according to the orthologs in Arabidopsis

According to the bioinformatics analysis of the HD-ZIP gene family, it has been found that the *PavHB16* belongs to the HD-ZIP I subgroup, and it has four motifs. To learn more genes that regulate plant organ shedding, the expression pattern of the HD-ZIP gene family was analyzed in different tissues (Fig. 12 a). The semiquantitative and qRT-PCR data showed that *PavHB16* and *PavHB18* were significantly up-regulated in flowers, abscising carpopodium (two different developmental stages), old petioles, and the old leaves (Fig. 12 b). Moreover, the qRT-PCR result also proves this (Fig. 12 c). These results suggest that *PavHB16* and *PavHB18* may contribute to the plant organ abscission of sweet cherry. Additionally, the protein-protein interaction network result showed that the *PavHB16* interact with abscisic acid-responsive genes (Fig. 13 a), and the *PavHB18* interacted with beta-glucosidase 24 (Fig. 13 b). These results showed that the genes, *PavHB16* and *PavHB18* could play a crucial role in the sweet cherry organ abscission.

## Discussion

In the present study, we combined the morphology, transcriptome, proteome, phytohormone and HD-ZIP gene family profiling to reveal the molecular mechanism of sweet cherry fruitlet abscission. Our research reveals that the abscission of sweet cherry fruitlet might be associated with the plant hormone biosynthesis and signal transduction, cell wall remodeling, cytoskeleton, and transcription factor. Interestingly, we found a transcription factor that may be related to sweet cherry flowers, leaves, and fruitlet. These results are similar to those of previous studies [16, 26, 30, 33]. Understanding the shedding of plant organs at the molecular level is of great significance for sparse flowers and fruitlet and mechanical picking.

### Pollen abortion leads to fruitlet shedding

The healthy embryo development, renders the fruit hormone in a relatively stable state, thus promoting further fruit growth. On the contrary, embryo abortion will often lead to the shedding of the fruitlet [34]. However, the embryo’s natural development relies heavily on successful fertilization [35]. Failed fertilization will lead to embryo abortion, which will trigger hormone imbalance and fruitlet shedding. Numerous studies have found that the regulation of endogenous hormones is closely related to the embryonic development of plants [36]. In this study, the sweet cherry ‘Santina’ is a Self-incompatibility variety. Therefore, the pollen vigor of its pollination tree ‘Brooks’ plays a vital role in embryo development. The result found that the pollen germination rate of ‘Brooks’ was just 15.56%.

Moreover, the pollen morphology showed that most of the pollen was deformed. This trend indicates that sweet cherry embryo abortion may be affected by irregular pollen from pollinating trees. Prior research has shown premature fruit drop can be caused by several factors, including lack of pollination or embryonic degeneration [37]. Therefore, for self-incompatible fruit trees, the normal development of pollinated tree pollen plays an important role in increasing the fruit setting rate.

### Phytohormones involve in the fruitlet abscission of sweet cherry

The regulatory effects of phytohormones are of critical importance in the entire plant organ abscission process, as they mediate plant organ responses to stress [7]. Phytohormones perhaps play a role in promoting or inhibiting shedding signals depending on the different tissues, the concentrations, the homeostasis and the affinities of their receptors, transport or their interactions with each other, and responses are complex [38]. Several phytohormones, including ethylene (ETH), abscisic acid (ABA), jasmonic acid (JA), and methyl jasmonate (MeJA), act as abscission-accelerating signals [7, 39], while auxin, gibberellins (GA), cytokinin (CTK) and polyamines are considered as abscission inhibitors [7, 40]. Since phytohormones are involved in the entire plant development cycle, several genes that control abscission also form part of the biosynthetic phytohormone and signal transduction pathways, or influence their metabolism [12, 41]. Though the roles of the many phytohormones remain unclear, auxin/ethylene balance and ABA have been proved to touch off shedding [42]. At present, the concentrations of auxin in abscising carpopodium was substantially lower than that of retention carpopodium.

Moreover, increased abscisic acid may also be responsible for regulating the shedding of carpopodium. However, the decreased ACC does not reflect the ethylene lessened concentrations. Although the ACC concentrations lower in the abscising carpopodium, If the 1-aminocyclopropane-1-Carboxylate Oxidase (ACO) content increases, it would lead to an increase in ethylene concentration [16]. Therefore, the ACO plays a vital role in the ethylene biosynthesis, and the transcriptome result showed that the ACO was up-regulated. Additionally, the reduction of auxin content can increase the sensitivity of the abscission zone to ethylene [43]. Hence, once the auxin content in the abscission zone decreases, the sensitivity of the abscission zone to ethylene will increase, and even a little of ethylene will trigger shedding [44]. Therefore, auxin content in the abscission zone plays a vital role in regulating shedding.

### Transcription factors regulate fruitlet abscission of sweet cherry

Multiple genes regulate the shedding of plant organs, and transcription factors also play a critical role [18, 25, 27]. In the shedding of the tomato flower organ, the *jointless*, which is the MADS-box gene family plays an important role in the differentiation of the abscission zone [45]. Also, during the process of litchi fruitlet abscission, when fruitlet abscission begins or is induced by girdling plus defoliation (GPD) or ethylene (ETH) treatment, *LcHB2/3* was induced, which then accelerate the biosynthesis of ethylene and abscisic acid by directly binding to the promoters of *LcACO2/3*, *LcACS1/4/7*, and *LcNCED3* genes [42]. In the current study, some transcription factors of the MADS family have also been significantly up-regulated, which may also play a necessary role in the differentiation of the abscission zone. The most noteworthy is the genes of the homeobox family, especially *PavHB16* and *PavHB18*, which belong to the HD-ZIP I subfamily and had a close relationship with *LcHB2* in litchi [42]. Moreover, according to sequence alignment analysis, the promoter of the sweet cherry gene *PavCEL* gene of sweet cherry and the promoter of the *LcCEL2* of litchi have the same sequence (AAATTAAA) that can be combined with the *LcHB2* (Additional file 11). Therefore, it can be speculated that the genes of the HD-ZIP gene family play an extensive role in regulating the shedding of sweet cherry.

### Cell wall remodeling implicates in fruitlet abscission of sweet cherry

The remodeling of cell walls accompanies the shedding of plant organs, so genes related to cell wall remodeling are particularly important in promoting the shedding of plant organs. The genes of cellulase, pectinase, polygalacturonase, expansin, xyloglucan endotransglucosylase/hydrolase, and peroxidase were found to be significantly up-regulated during the abscission cycle of citrus, litchi and tomatoes [2, 45, 46]. Additionally, in litchi, it was demonstrated that *LcCEL2* and *LcCEL8* could be regulated by *LcHB2*, thereby promoting the litchi shedding [47]. Our findings found that several genes linked to cell wall remodeling were significantly up-regulated in the abscising carpopodium that was about to shedding. The explanation why these cell wall modifying enzyme genes can be related to the shedding of plant organs is that each protein they encode has a pivotal function. Cellulase’s primary function is to hydrolyze cellulose, which is the major cell wall constituent [48]; both pectinase and polygalacturonase are involved in the hydrolysis of pectin [49]; Xyloglucan endotransglucosylase/hydrolase mainly hydrolyzes the hemicellulose in the cell wall, and the expansin plays a fatal role in the ripening and softening of the fruit [21]; Besides, peroxidase plays a crucial role in the synthesis of lignin; in the study of plant organ shedding, although the relationship between the accumulation of lignin and the shedding of plant organs has not been found, it was found that lignin is produced in the abscission zone of other plants [21]. This evidence showed that peroxidase plays an important role in promoting lignin synthesis. Therefore, up-regulated peroxidase gene expression can play a positive role in the plant organ shedding. In recent research, the genes of cellulase, pectinase, polygalacturonase, expansin, xyloglucan endotransglucosylase/hydrolase, and peroxidase were found to be significantly up-regulated in the abscising carpopodium. Besides, previous proteomic studies have also shown the same results [50]. These results suggested that the remodeling of cell walls might promote the sweet cherry fruitlet shedding.

### The cytoskeleton regulate fruitlet abscission of sweet cherry

The cytoskeleton is a fundamental component of a cell and plays a crucial role in the entire cell development process. Furthermore, the pectin transport and its modifying proteins occur principally through the actin cytoskeleton. Therefore, it is unsurprising that defects in actin filament organization affect cell adhesion. The Actin-related protein2/3 complex (Arp2/3) is highly conserved and is the prime component in regulating branching and nucleation of actin filaments [51]. Additionally, the microtubule skeleton is the basic skeleton that constitutes a cell [52]. And there have been studies that plant organ shedding has also been associated with the arrangement of microtubules [44]. In this study, some cytoskeleton-related genes, including α-TUB, MAP were down-regulated, which maybe one of the reasons regulate the sweet cherry fruitlet abscission. The result implies that the formation of plant cells is hindered, which in turn leads to programmed cell death and the shedding of plant organs. Also, the proteomics at the early stage of this research group also showed that a close relationship existed between the cytoskeleton and the shedding of sweet cherry fruit [50].

## Conclusion

**Fig. 14:**
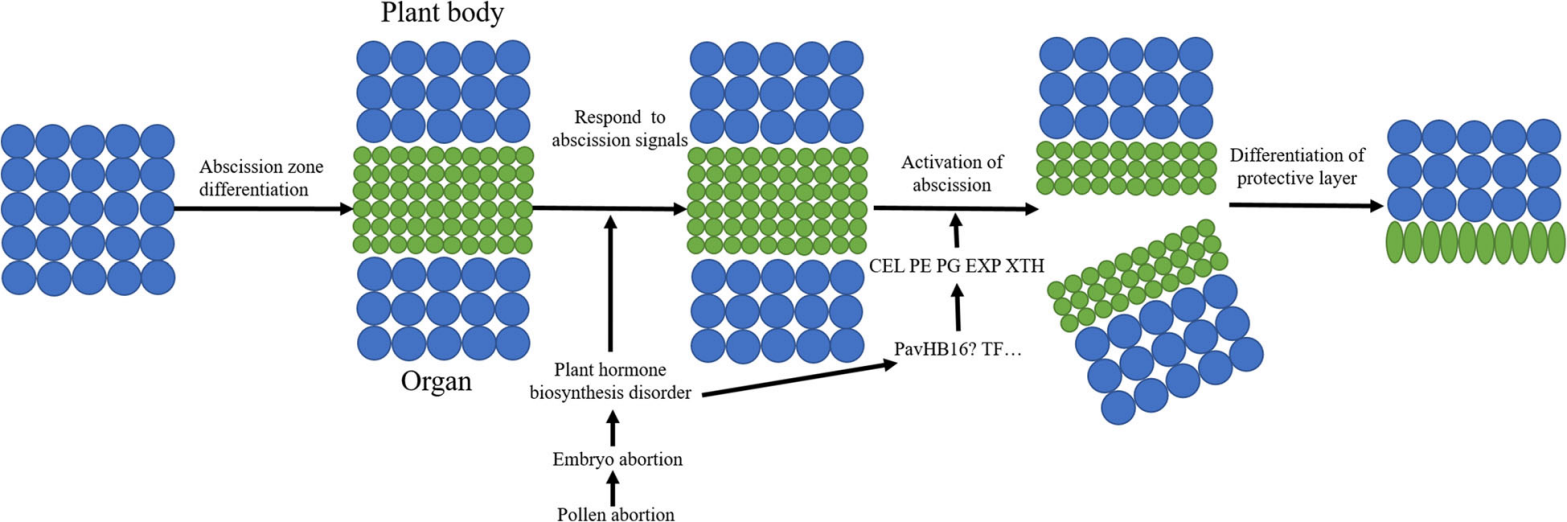
Model of fruitlet abscission in sweet cherry

The shedding of plant organs is caused by a range of factors. Sweet cherry will abscission during their over-maturity, it is essential to distinguish the abscission zone during the development phase. However, owing to the pollination tree’s malformed pollen, pollen tube germination is restricted, and normal pollination and fertilization could not be completed, which led to the abortion of the embryo. The embryo abortion causes imbalances in plant hormones, such as raised ethylene production and decreased auxin production. Reduced auxin content will increase the sensitivity of the abscission zone to ethylene, thereby activating the expression of transcription factors related to plant organ shedding in cells. The transcription factors were translated into proteins and then bound to the promoters of genes, which can degrade the cell wall. After that, the cell wall degradation-relate genes, such as *CEL* and *PG*, were initiated, thereby increasing the content of these cell wall degrading enzymes. Under the appropriate pH and temperature, these enzymatic activity rises, consequently degrading the middle lamella and cell wall, which persuade sweet cherry fruit to drop. It is worth noting that the transcription factors of the HD-ZIP gene family may play an indispensable role in regulating the shedding of plant organs. In the future, the *PavHB16*, *PavHB18* and other transcription factors will be studied in detail, to further reveal the molecular regulation mechanism of sweet cherry fruitlet shedding (Fig. 14).

## Methods

### Plant materials

The sweet cherry (‘Santina’) trees, grown in Weining County, Guizhou Province, China (E:104.12, N: 27.25) was used as a primary material. Three trees with similar growth vigor were selected, and abscising carpopodium and non-abscising carpopodium were taken 20 days after flowering. This carpopodium were quickly frozen in liquid nitrogen and brought back to the laboratory for storage in a − 80 °C refrigerator. The ‘Brooks’, which was used as the pollination tree for ‘Santina’ since the latter characterizes in self-incompatibility. It has also grown in Weining County, Guizhou Province, China (E:104.12, N: 27.25), and the pollen was used to detect the pollen viability and morphology characters. Three ‘Qianying No 1’ trees, grown in Xiuwen County, Guizhou Province, China (E: 106.61, N: 26.85) were used, and the pollen of ‘Qianying No 1’ was used to detect the viability and morphology characters as a control. For tissue-specific expression pattern analysis, various sweet cherry ‘Santina’ tissues were used. Each tree was deemed a biological replicate of each sample, three replicates were performed.

### Determination of pollen germination ratios and morphology characters

In vitro pollen germination was performed according to previously published methods with slight modification [53], the pollen germination rate was determined by in vitro culture. The medium formula was: MS medium + 10% sucrose + 0.5% agarose + 0.1 g/L of boric acid. Briefly, dropping the medium in the groove on the glass slide, after it was solidified, the pollen was sprinkled on the medium with a dissecting needle, placed in a petri dish with moist filter paper and covered, and placed at 25 °C incubators. Four hours later, the pollen germination rate was determined after microscopic observation. The pollen tube length was greater than the pollen grain diameter, which was recorded as germination, otherwise, it was recorded as no germination. Further, as well as three biological replicates per sample and each replicate were randomly selected for 3 fields of view, and the number of pollens per field was counted. Percentage pollen germination was calculated using the formula:

$$ \mathrm{Pollen}\ \mathrm{germination}\ \mathrm{rate}\ \left(\%\right)=\frac{\mathrm{Number}\ \mathrm{of}\ \mathrm{germinated}\ \mathrm{pollen}}{\mathrm{Total}\ \mathrm{number}\ \mathrm{of}\ \mathrm{observed}\ \mathrm{pollen}}\times 100\%. $$

Around 150 flowers were selected in three replications of each cultivar at the late balloon phase and placed in paper bags. (one replication included 50 flowers from all sides of three sweet cherry trees). Anthers were placed in kraft paper and dried at room temperature until burst and pollen dusting. Pollen grains were placed on the double-sided transparent tape mounted on the microscope stage by dissecting needle. Pollen samples were coated with 0.02 μm gold alloys in a sputter-coater BAL-TEC SCD 005 and monitored at 15 kV with a JEM-2100 (UHR). According to the morphological characteristics of pollen, it can be divided into two types. Namely, the normal pollen, which is oval and plump, as well as the malformed pollen is irregular and withered. Three biological replicates per sample and each biological replicate were randomly selected for 3 fields of view, and the number of pollens per field was counted. Percentage pollen was calculated using the formula: malformed pollen (%) = $\frac{\mathrm{Malformed}\ \mathrm{pollen}}{\mathrm{Total}\ \mathrm{number}\ \mathrm{of}\ \mathrm{observed}\ \mathrm{pollen}}\times 100\%$.

### Comparison of morphological characters and determination of the embryo weight

According to the morphological characteristics, these morphological traits were described amid abscising and non-abscising fruitlet. Additionally, ten abscising fruitlet and ten non-abscising fruitlet were taken, later the fruitlet were dissected, and the weight of their embryos was measured by electronic micro-balance with five biological replicates (A total of 50 embryos per sample). The significance was determined by the student’s t-test (*P* < 0.05) with SPSS 21.

### Extraction and determination of hormone levels

The contents of, IAA, IBA, GA3, GA4, GA7, ABA, ACC, JA, and MeJA in abscising and non-abscising carpopodium were determined by high-performance liquid chromatography-tandem mass spectrometry (HPLC-MS/MS), three biological replicates each sample. The method of extraction and content determination of hormones in this study was used previously published [54]. Finally, the significance was determined by the student’s t-test (*P* < 0.05).

### Total RNA extraction, Illumina library construction and sequencing

By using the RNA prep Pure Polysaccharide Polyphenol Plant Total RNA Extraction Kit (Tiangen, Beijing, China) total RNA was extracted and the DNA was eliminated. Three biological replicates were performed. RNA quality and concentration were measured using a Nanodrop 2000 micro-spectrophotometer (Thermo Fisher Scientific, Waltham, MA, USA), and agarose gel electrophoresis. The integrity of RNA was assessed using an Agilent 2100 bioanalyzer (Agilent Technologies). The mRNA was enriched using magnetic beads with Oligo (dT) and cleaved into short fragments (~ 200 nt) in a fragmentation buffer. The reverse transcription was conducted with a random hexamer primer and then the second-strand cDNA was synthesized. After end repair, the 5′ tails were phosphorylated, the 3′ tails were added with an adenine. Sequencing adaptors were ligated to the double-stranded DNA fragments. Afterward, the fragments were amplified by PCR to construct the cDNA library. The library was sequenced using the Illumina cluster station and Illumina HiSeq 4000 sequencing platform.

### Bioinformatics analysis of RNA-Seq data

The raw reads were obtained from each cDNA library. To obtain clean reads, the SOAPunke and trimmomatic software was used to remove the adaptor sequences, low-quality reads and reads containing more than 5% unknown bases. The genome (Prunus_avium_v1.0.a1) of sweet cherry from the Genome Database for Rosaceae (GDR) (https://www.rosaceae.org/species/prunus_avium/genome_v1.0.a1) was used as the reference and for transcript annotations. The gene expression levels were calculated normalized to FPKM (Fragments Per Kb per Million reads). Differentially expressed genes (DEGs) were identified using the DEseq2 R package, and the commands could be found in Additional file 12. The resulting *p-values* were adjusted using the Benjamini and Hochberg’s approach to controlling the false-discovery rate (FDR). The significance of the DEGs was determined with a *p-value* < 0.05 found by DEseq2. Transcripts with log 2 (Fold Change) > 1 and *p-value* < 0.05 were considered significant differential expression. The gene ontology (GO) and Kyoto Encyclopedia of Genes and Genomes (KEGG) enrichment analyses of the DEGs were implemented using omicshare (https://www.omicshare.com/tools/), which is a bioinformatics platform. The proteomic data published in the early stage of this research group was used to analyze the correlation with the transcriptome [50]. The differentially accumulated proteins (DAPs) with 1.5-fold and differentially expressed genes with 2 folds are used for cooperative investigation.

The expression levels of samples under the various plant tissues, including abscising and non-abscising carpopodium, were validated by qRT-PCR using PowerUp SYBR Green Master Mix (ThermoFisher, Chongqing, China) in a volume of 10 μL, containing 5 μl of SYBR Green Master Mix, 100 ng of cDNA template, and 0.5 μM of each of the forward and reverse primers. The qRT-PCR amplification was performed as follows: 95 °C for 30 s, followed by 40 cycles of 95 °C for 5 s and 60 °C for 30 s. The *PavEF-1α* and *PavRSP3* were used as internal control, and the primer sequences are listed in Additional file 13: Table S9. The relative gene expression level was executed using the 2^-ΔCt^ method [55] with the CFX Connect Real-Time PCR Detection System (Bio-Rad Laboratories, CA, USA). All other validations were performed in three replicates, both biological and technical.

### HD-ZIP gene family bioinformatics and expression levels analysis

A Hidden Markov Model (HMM) profile of the HD-ZIP domain (protein family ID: PF00046) was downloaded from Pfam (http://pfam.xfam.org/), and used as the query to search against a previous assembly sweet cherry genome database (https://www.rosaceae.org/species/prunus_avium/genome_v1.0.a1) using the HMMER 3.0 software (http://hmmer.org/). Furthermore, the obtained sequences were uploaded for the analysis of conserved domains to the SMART and Pfam websites. Proteins containing both the homeobox domain (HD, PF00046) and the homeobox-associated leucine Zipper domain (HALZ, PF02183) are classified as candidate proteins. A phylogenetic tree was constructed with MEGA (version 7.0) using the neighbor-joining method. Motif location was analyzed using MEME (version 4.12.0) (http://meme-suite.org/tools/meme). The expression levels were analyzed using semi-quantitative and quantitative Real-time PCR (qRT-PCR) methods, The *PavEF-1α* and *PavRSP3* were used as internal control, and the primer sequences were listed in Additional file 14: Table S10. The functional interacting networks of *PavHB16* and *PavHB18* are integrated into STRING (version 11.0).

## Supplementary Information

**Additional file 1: Table S1.** The germination rate and deformity rate of sweet cherry pollen.

**Additional file 2: Table S2.** The content of plant hormone between the abscising carpopodium and non-abscising carpopodium.

**Additional file 3: Table S3.** Summary of reads number from abscising carpopodium and non-abscising carpopodium.

**Additional file 4: Figure S1.** The Pearson correlation between samples.

**Additional file 5: Table S4.** Differentially expressed genes between abscising carpopodium and non-abscising carpopodium.

**Additional file 6: Table S5.** The enriched KEGG pathways of DEGs.

**Additional file 7: Table S6.** GO classification of DEGs.

**Additional file 8: Table S7.** The plant cell wall remodeling related gene expression levels.

**Additional file 9: Table S8.** Validation of RNA-seq results via qRT-PCR.

**Additional file 10.** The original gel images of internal genes and HD-ZIP gene family.

**Additional file 11. **The sequence of *PavCEL* promoter.

**Additional file 12.** The command of differentially expression genes identified by the DESeq 2 R package.

**Additional file 13: Table S9.** Primers for transcriptome data verification by qRT-PCR analysis in carpopodium.

**Additional file 14: Table S10.** A list of primer sequences of the 27 selected HD-ZIP genes for expression levels analysis.

## Data Availability

Raw sequence reads of the transcriptomes of abscising carpopodium and non-abscising carpopodium were deposited in the NCBI Sequence Read Archive (SRA) with accession numbers PRJNA636209.

## Abbreviations

ABA: Abscisic acid
ACC: 1-aminocyclopropane-1-carboxylatewere
ACO: 1-aminocyclopropane-1-carboxylate oxidase
AE: Abscising fruit embryo
AF: Abscising fruit
ARF: Auxin response factor
AUX/IAA: Auxin/Indole-3-Acetic
AUX1: Auxin efflux carrier
AZ: Abscising carpopodium abscission zone
CA: Abscising carpopodium
CN: Non-abscission carpopodium in the first stage
CTK: Cytokinin
DAF: Days after flowering
EG: Endoglucanase
EIN3: Ethylene insensitive 3
ERF: Ethylene response factor
EXP: Expansin
FA: Abscising fruit in the first stage
Fb: Flower bud
FL: Flower
FN: Non-abscising fruit in the first stage
FPKM: Fragments Per Kb per Million reads
GA: Gibberellin
HPLC-MS/MS: High-performance liquid chromatography-tandem mass spectrometry
IBA: Indole-3-butyric acid
JA: Jasmonic acid
KEGG: Kyoto Encyclopedia of Genes and Genomes
MeJA: Methyl jasmonate
NAF: Non-abscising fruit
NAZ: Non-abscising carpopodium abscission zone
NE: Non-abscising embryo
IAA: Indole-3-acetic acid
NPR5: Non-expressor of PR5
PAL: Pectin acetylesterase
PAO: Polyamine oxidase
PEM: Pectin methylesterase
PG: Polygalacturonase
PL: Pectate lyase
POD: Peroxidase
SAUR: Small auxin up RNA
St: Stem
TZ: Trans-zeatin
XTH: Xyloglucan endotransglucosylase/hydrolases
α-Trp: Tryptophan synthase alpha chain
β-gal: Beta-galactosidase
β-glc1: Beta-glucanase
