# Supplementary material for: Cross-talk between transcriptome, phytohormone and HD-ZIP gene family analysis illuminates the molecular mechanism underlying fruitlet abscission in sweet cherry (Prunus avium L)
Source: BMC Plant Biol. 2021 Apr 10;21:173. doi: 10.1186/s12870-021-02940-8 (PMC8035788; doi:10.1186/s12870-021-02940-8)
Supplement: Supplementary file 1 — Additional file 1: Table S1. The germination rate and deformity rate of sweet cherry pollen. [file 12870_2021_2940_MOESM1_ESM.docx]

Table S1 The germination rate and deformity rate of sweet cherry pollen

| Variety | Germination rate (%) | Deformity rate (%) |
| --- | --- | --- |
| Qianying No. 1 | 65.36% | 13.33% |
| Brooks | 15.56% | 88.81% |
