# Supplementary material for: Cross-talk between transcriptome, phytohormone and HD-ZIP gene family analysis illuminates the molecular mechanism underlying fruitlet abscission in sweet cherry (Prunus avium L)
Source: BMC Plant Biol. 2021 Apr 10;21:173. doi: 10.1186/s12870-021-02940-8 (PMC8035788; doi:10.1186/s12870-021-02940-8)
Supplement: Supplementary file 2 — Additional file 2: Table S2. The content of plant hormone between the abscising carpopodium and non-abscising carpopodium. [file 12870_2021_2940_MOESM2_ESM.docx]

Table S2 The content of plant hormone between the abscising carpopodium and non-abscising carpopodium

|  | CA1 | CA2 | CA3 | CA mean | CN1 | CN2 | CN3 | CN mean |
| --- | --- | --- | --- | --- | --- | --- | --- | --- |
| IAA | 0.7109 | 0.6431 | 0.6163 | 0.6568 | 2.4557 | 2.2761 | 2.5163 | 2.4161 |
| IBA | 0.1107 | 0.1063 | 0.1395 | 0.1188 | 0.2869 | 0.3515 | 0.2752 | 0.3045 |
| GA3 | 0.2138 | 0.2622 | 0.2241 | 0.2334 | 0.4066 | 0.4496 | 0.4448 | 0.4337 |
| GA4 | 5.3136 | 5.4449 | 3.8723 | 4.8770 | 4.6639 | 4.5368 | 4.4506 | 4.5504 |
| GA7 | 0.0387 | 0.0518 | 0.0378 | 0.0428 | 0.1013 | 0.1116 | 0.0879 | 0.1003 |
| TZ | 0.0304 | 0.0272 | 0.0349 | 0.0309 | 5.0014 | 5.4310 | 4.9277 | 5.1200 |
| ABA | 28.6695 | 28.0921 | 29.4610 | 28.7408 | 22.2296 | 21.3842 | 22.5174 | 22.0437 |
| ACC | 652.7568 | 648.0335 | 689.9512 | 663.5805 | 782.2831 | 831.5152 | 850.9434 | 821.5806 |
| JA | 6.9820 | 6.9946 | 7.2151 | 7.0639 | 699.7722 | 700.9596 | 696.5816 | 699.1044 |
| MeJA | 0.1245 | 0.1144 | 0.1250 | 0.1213 | 1.2124 | 1.4589 | 1.6312 | 1.4341 |
| (TZ+GA3+IAA)/ABA | 0.0333 | 0.0332 | 0.0297 | 0.0320 | 0.3538 | 0.3814 | 0.3503 | 0.3615 |
