# Supplementary material for: Cross-talk between transcriptome, phytohormone and HD-ZIP gene family analysis illuminates the molecular mechanism underlying fruitlet abscission in sweet cherry (Prunus avium L)
Source: BMC Plant Biol. 2021 Apr 10;21:173. doi: 10.1186/s12870-021-02940-8 (PMC8035788; doi:10.1186/s12870-021-02940-8)
Supplement: Supplementary file 3 — Additional file 3: Table S3. Summary of reads number from abscising carpopodium and non-abscising carpopodium. [file 12870_2021_2940_MOESM3_ESM.docx]

Table S3 Summary of read numbers from abscising carpopodium and non-abscising carpopodium

| Sample name | CA1 | CA2 | CA3 | CN1 | CN2 | CN3 |
| --- | --- | --- | --- | --- | --- | --- |
| Total raw reads (M) | 43.24 | 47.80 | 55.38 | 50.35 | 52.17 | 59.04 |
| Total clean reads (M) | 42.71 | 47.16 | 54.62 | 49.46 | 51.27 | 58.04 |
| Clean read ratio (%) | 98.78 | 98.66 | 98.62 | 98.24 | 98.28 | 98.32 |
| Clean read q20(%) | 97.23 | 97.00 | 97.19 | 97.13 | 97.43 | 97.42 |
| Clean read q30(%) | 92.47 | 91.98 | 92.37 | 92.32 | 93.07 | 92.99 |
| Total mapping genome  reads (M) | 38.22 | 42.87 | 48.65 | 44.49 | 46.36 | 51.88 |
| Total mapping genome  Ratio (%) | 89.5 | 90.9 | 89.08 | 89.95 | 90.41 | 89.37 |
