# Supplementary figures and images for: Cross-talk between transcriptome, phytohormone and HD-ZIP gene family analysis illuminates the molecular mechanism underlying fruitlet abscission in sweet cherry (Prunus avium L)
Source: BMC Plant Biol. 2021 Apr 10;21:173. doi: 10.1186/s12870-021-02940-8 (PMC8035788; doi:10.1186/s12870-021-02940-8)

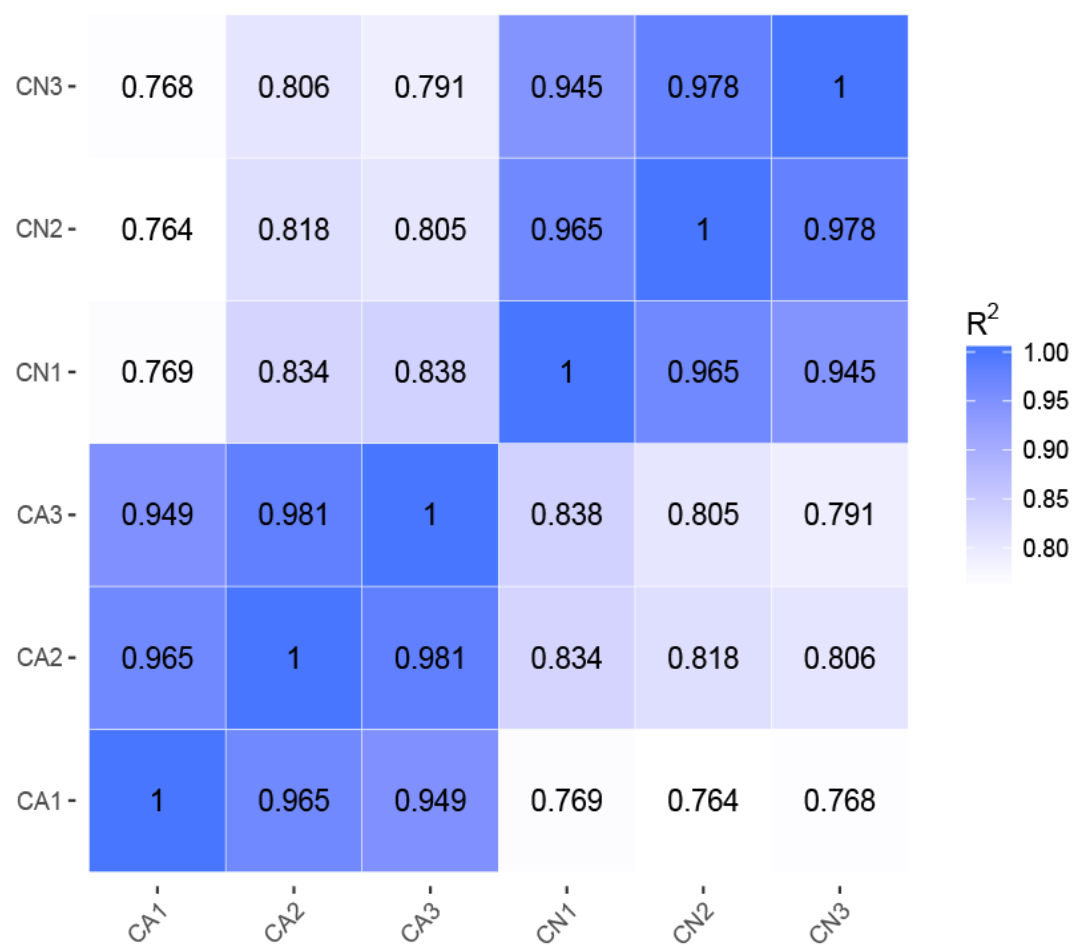

Figure S1 The pearson correlation between samples.

Supplement: Supplementary file 4 — Additional file 4: Figure S1. The Pearson correlation between samples. [file 12870_2021_2940_MOESM4_ESM.pdf]
