# Supplementary material for: Cross-talk between transcriptome, phytohormone and HD-ZIP gene family analysis illuminates the molecular mechanism underlying fruitlet abscission in sweet cherry (Prunus avium L)
Source: BMC Plant Biol. 2021 Apr 10;21:173. doi: 10.1186/s12870-021-02940-8 (PMC8035788; doi:10.1186/s12870-021-02940-8)

**Figure S2. The original gel images of refrence gene and HD-ZIP gene family.**

M: Maker; CA1: Abscising carpopodium in the first stage; CN1: Non-abscission carpopodium in the first stage; CA2: Abscising carpopodium in the second stage; CN2: Non-abscission carpopodium in the second stage; Fb: Flower bud; FL: Flower; FN1: Non-abscising fruit in the first stage; FN2: Non-abscising fruit in the second stage; FA1: Abscising fruit in the first stage; FA2: Abscising fruit in the second stage; Pe1: Young leaf petiole; Pe2: Old leaf petiole; Le1: Young leaf; Le2: Old leaf; St: Stem.

EF-1α


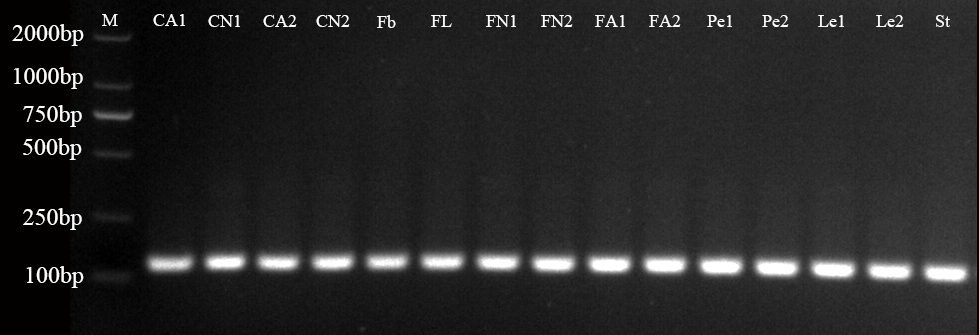


RSP3


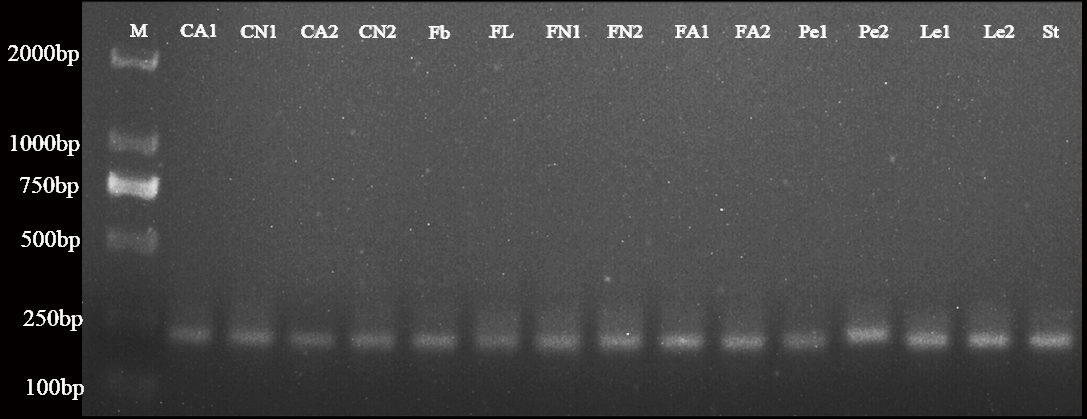


PavHB2


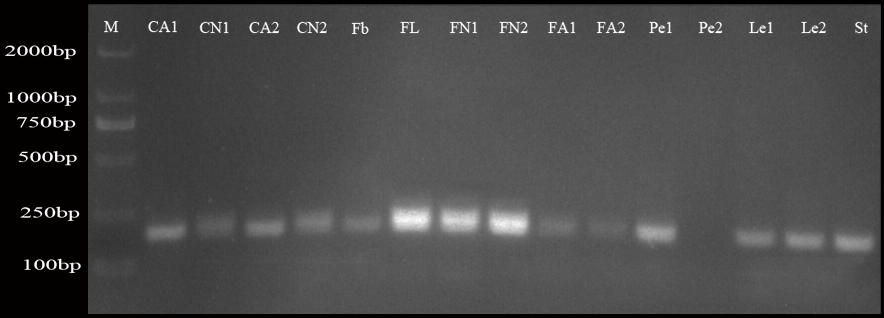


PavHB17


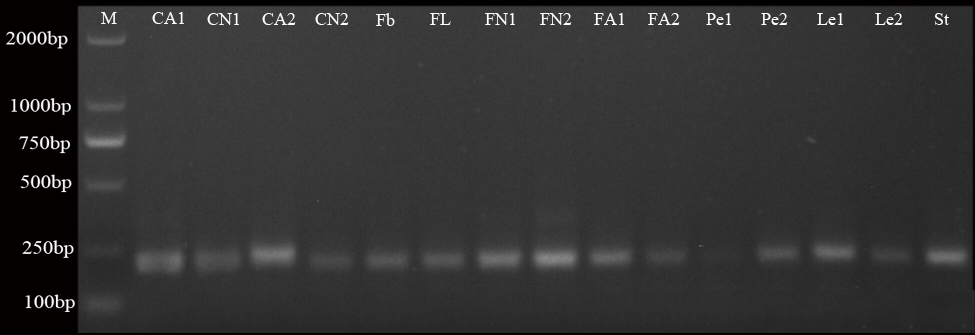


PavHB12


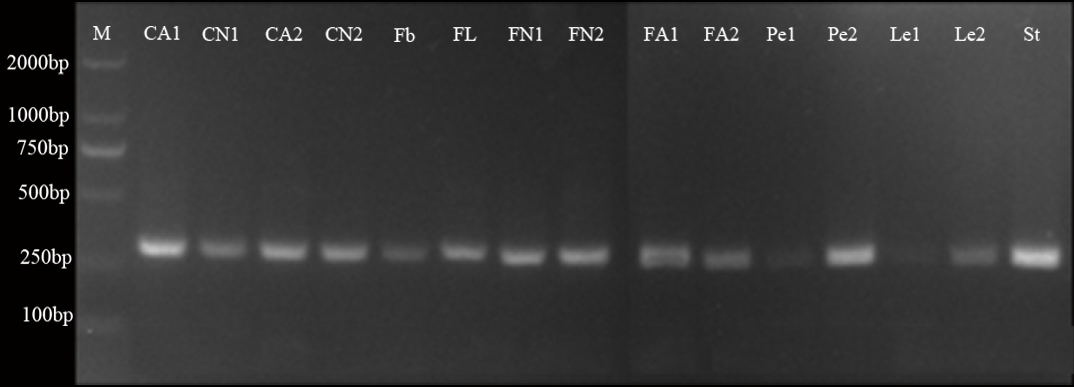


PavHB13


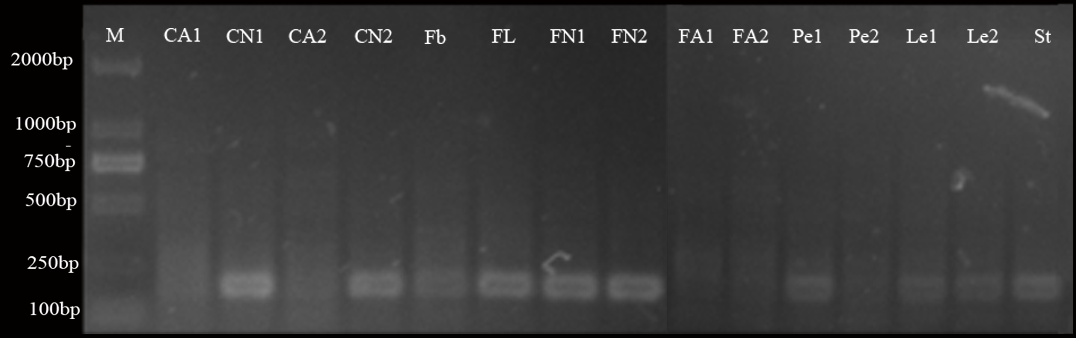


PavHB18


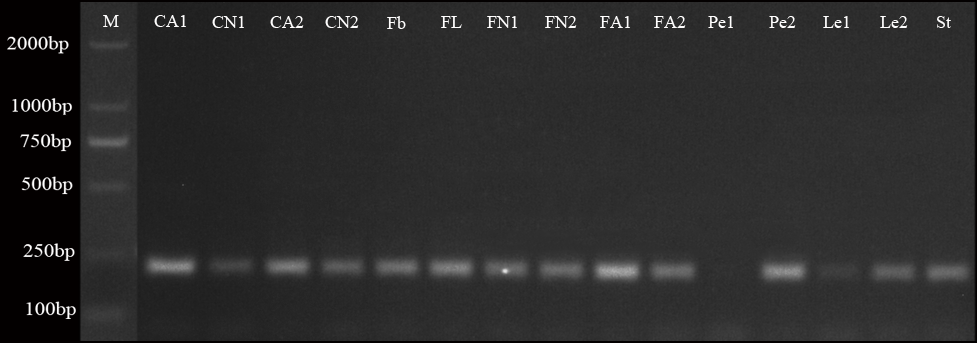


PavHB20


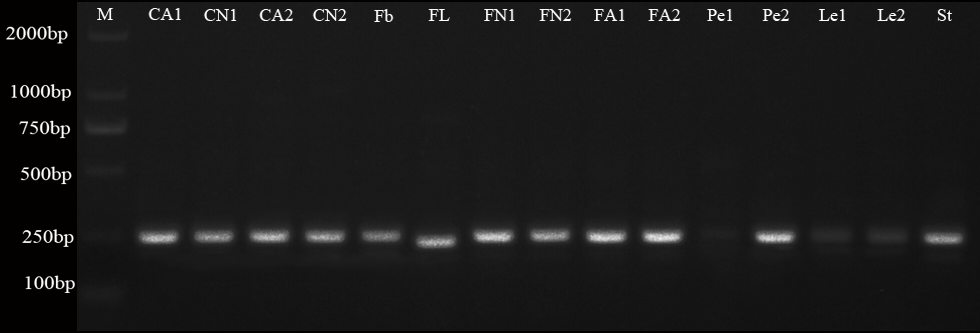


PavHB19


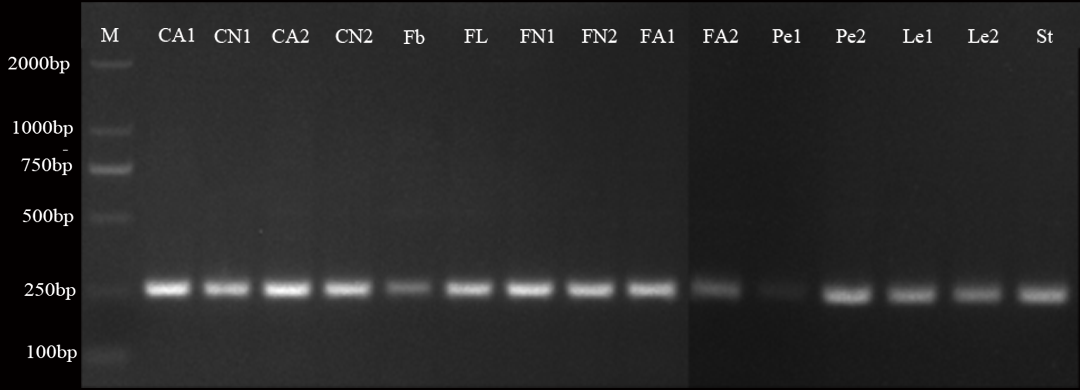


PavHB16


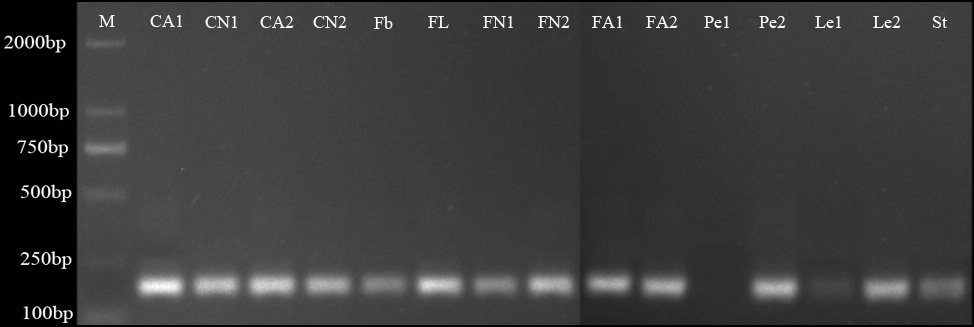


PavHB24


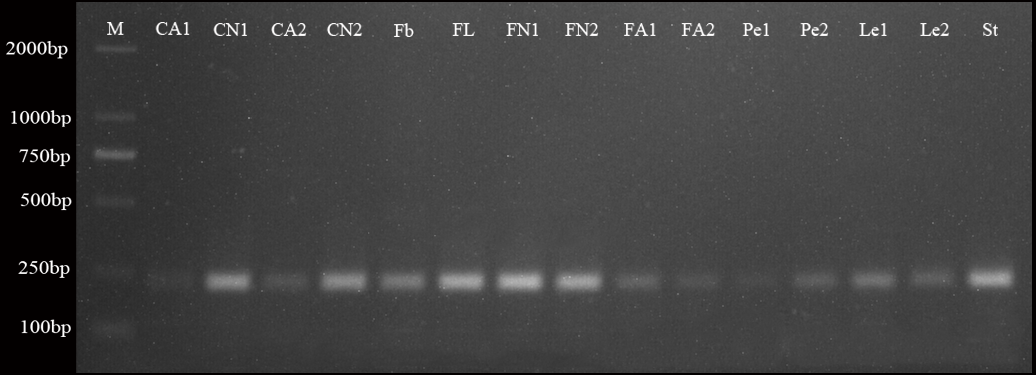


PavHB23


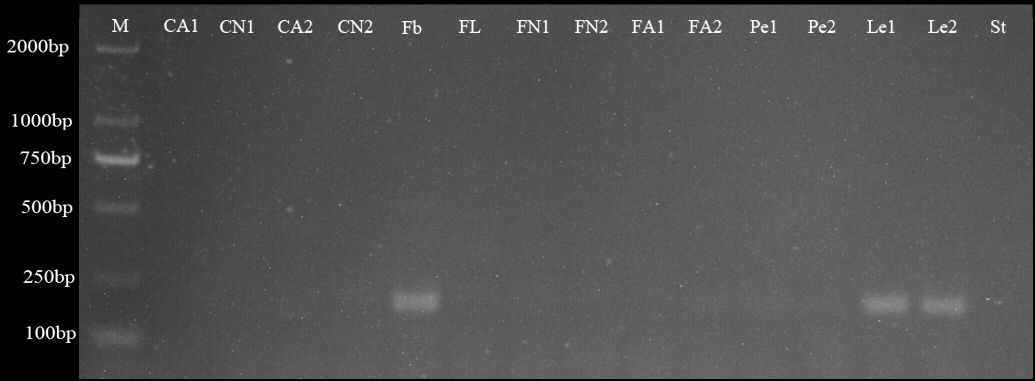


PavHB5


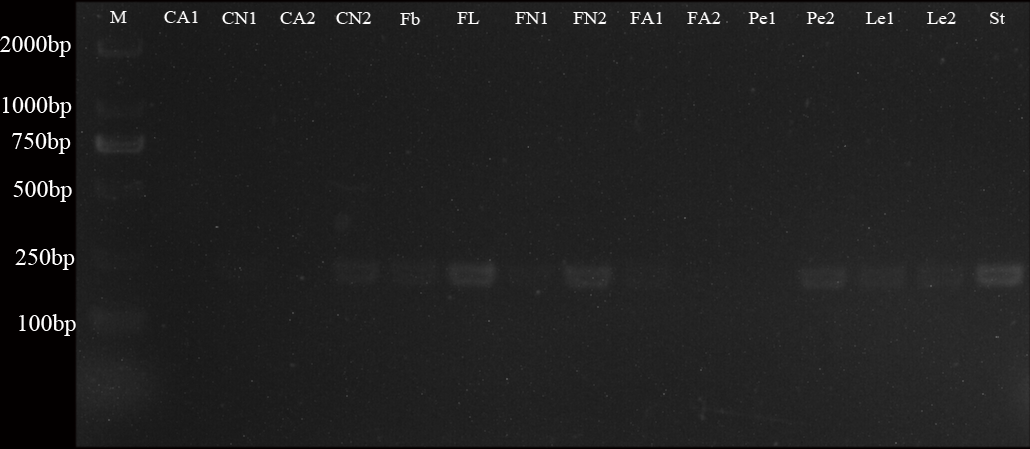


PavHB9


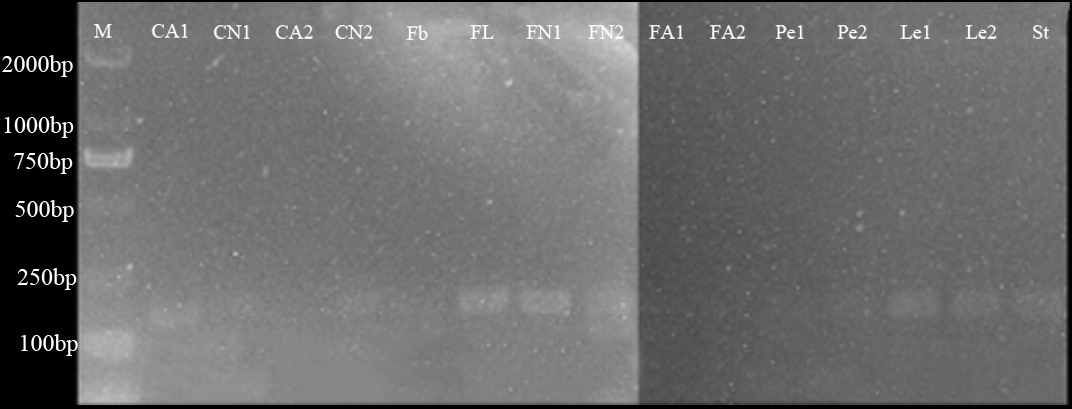


PavHB10


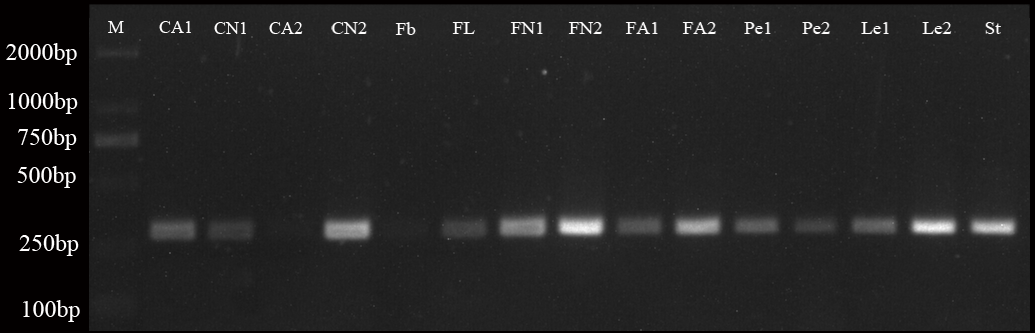


PavHB25


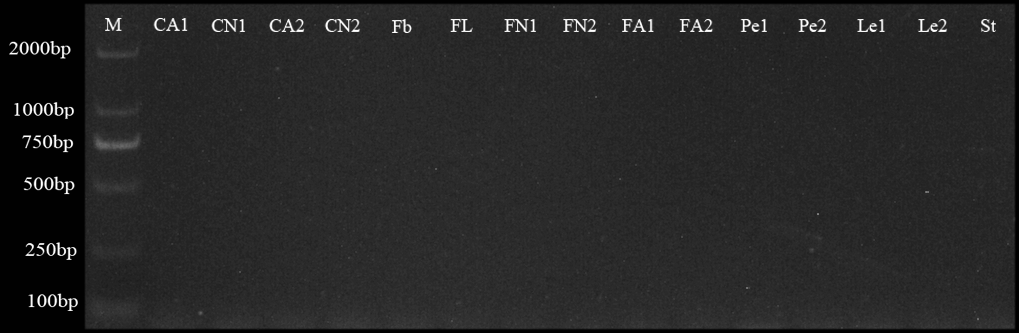


PavHB26


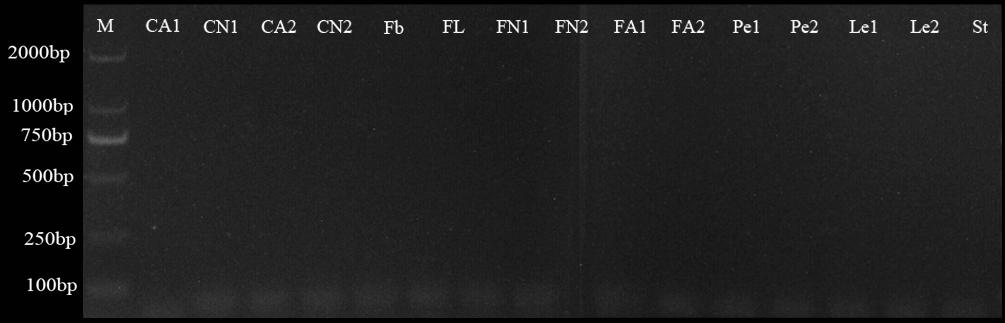


PavHB21


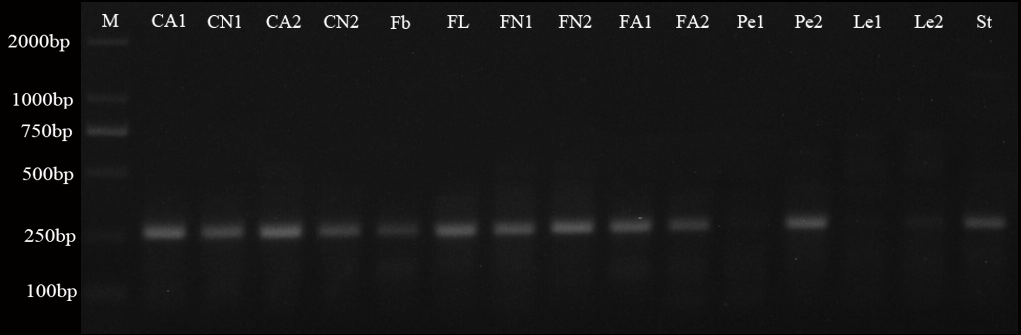


PavHB1


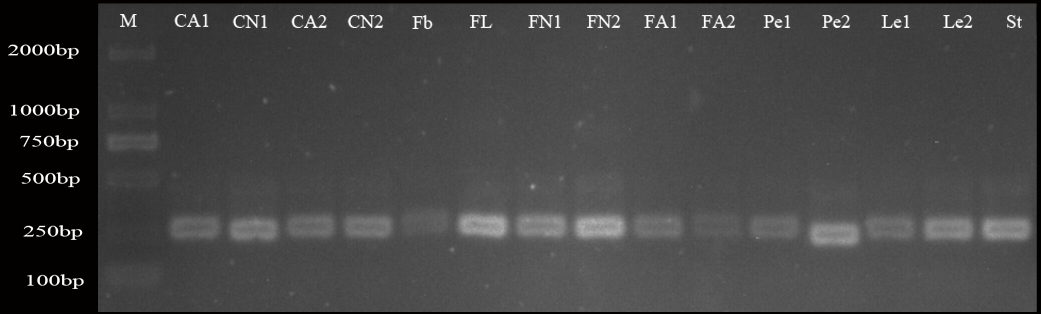


PavHB3


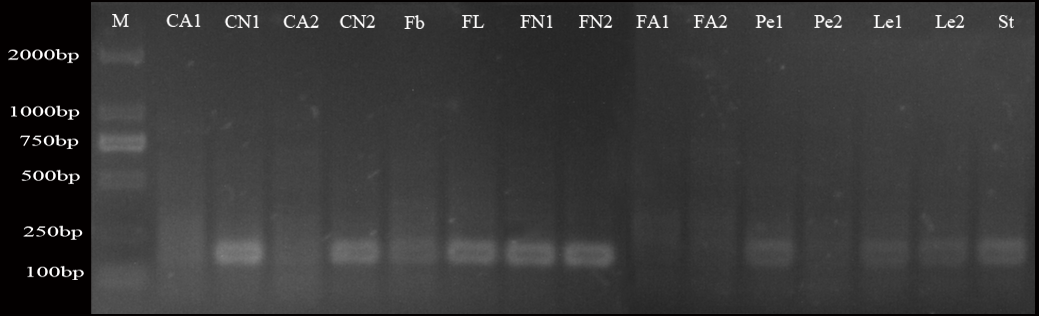


PavHB22


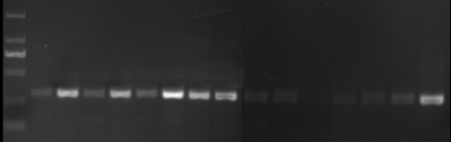


PavHB14


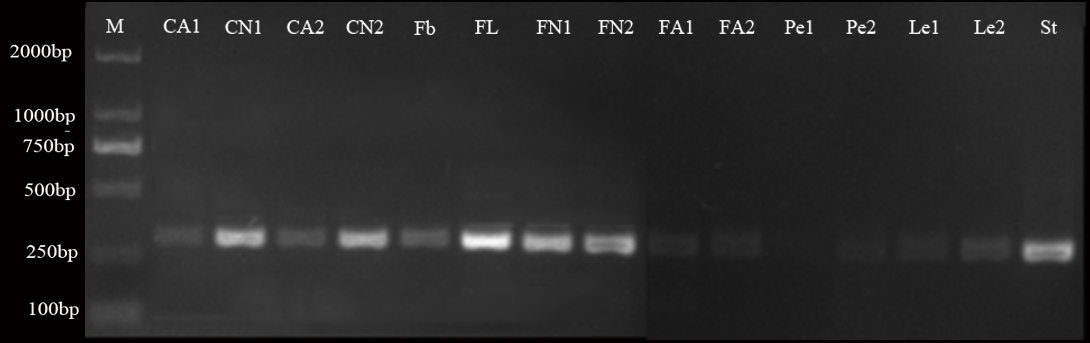


PavHB4


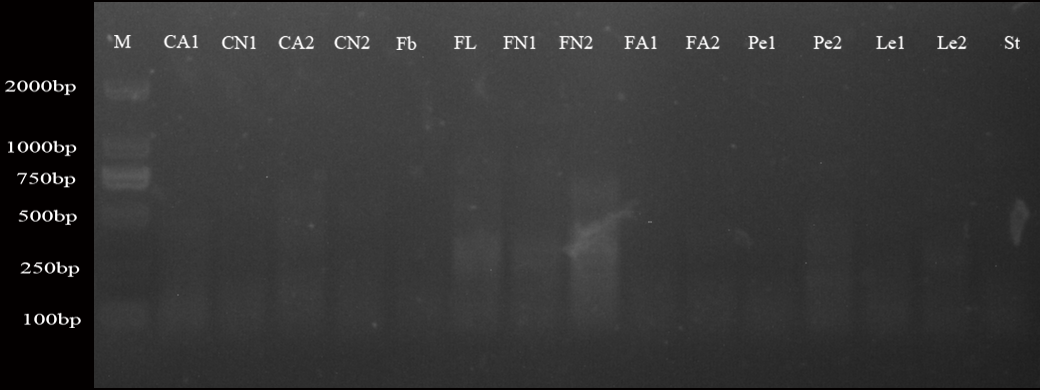


PavHB15


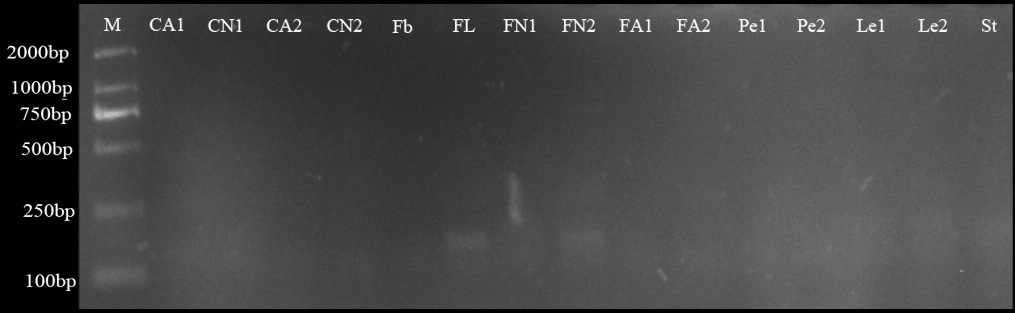


PavHB6


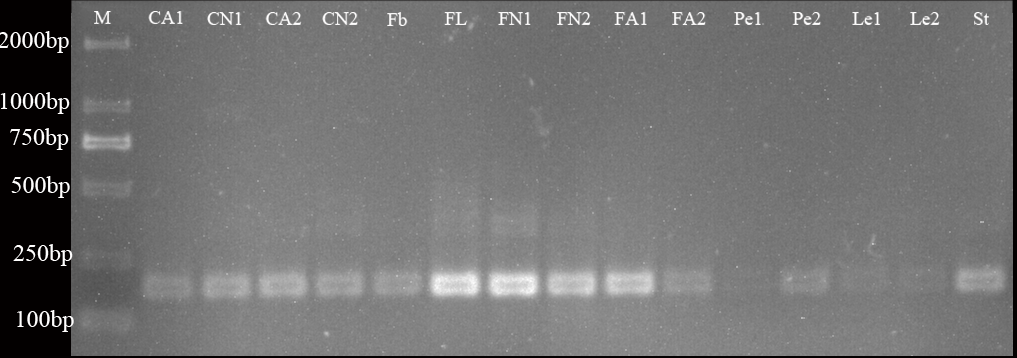


PavHB27


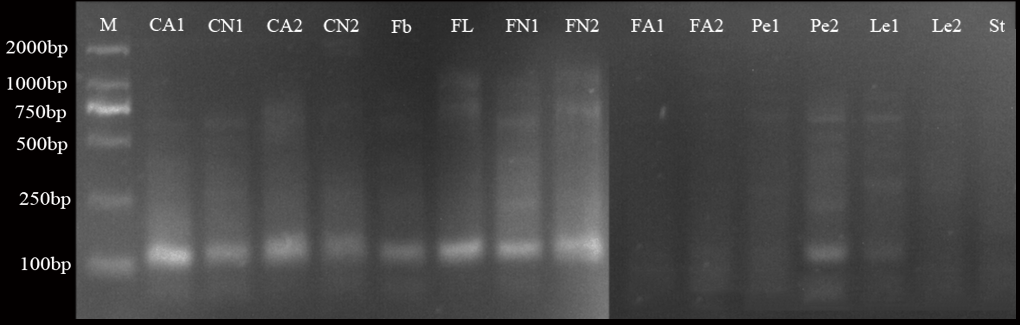


PavHB8


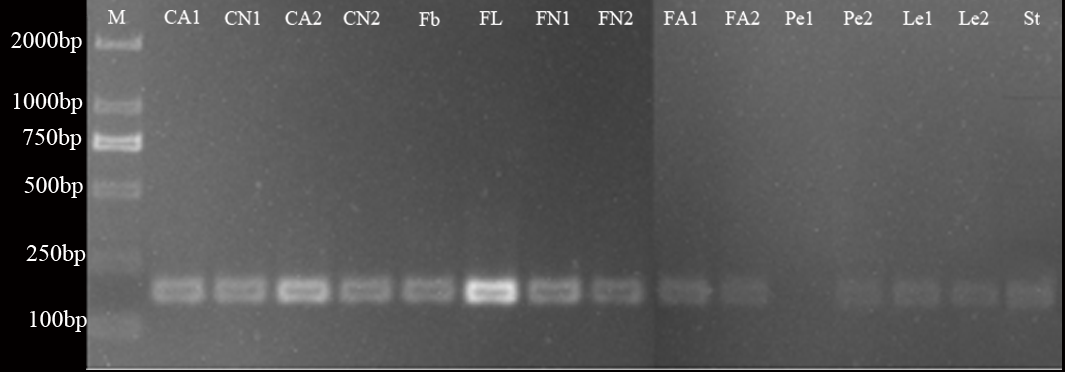


PavHB11


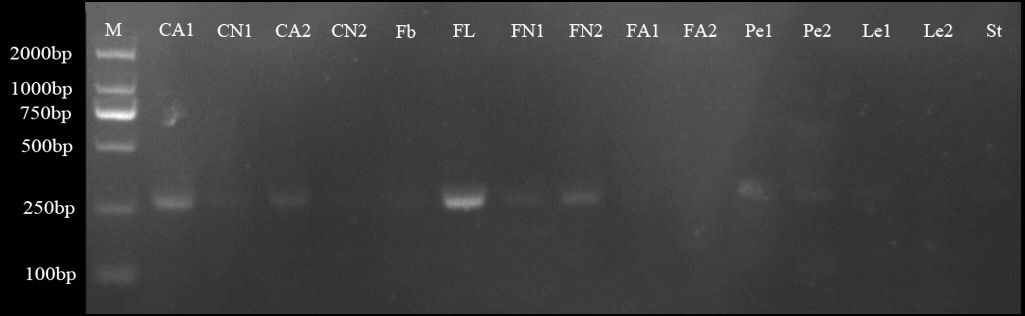


PavHB7


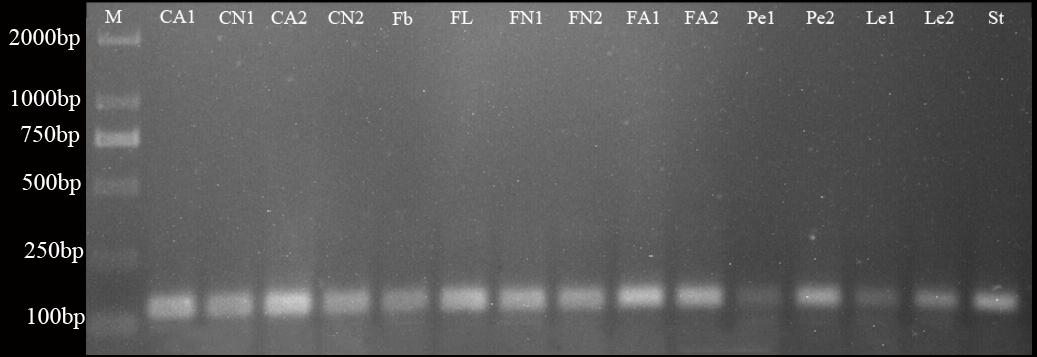

Supplement: Supplementary file 10 — Additional file 10. The original gel images of internal genes and HD-ZIP gene family. [file 12870_2021_2940_MOESM10_ESM.docx]
