# Supplementary material for: Cross-talk between transcriptome, phytohormone and HD-ZIP gene family analysis illuminates the molecular mechanism underlying fruitlet abscission in sweet cherry (Prunus avium L)
Source: BMC Plant Biol. 2021 Apr 10;21:173. doi: 10.1186/s12870-021-02940-8 (PMC8035788; doi:10.1186/s12870-021-02940-8)
Supplement: Supplementary file 11 — Additional file 11. The sequence of PavCEL promoter. [file 12870_2021_2940_MOESM11_ESM.pdf]

>Pav_sc0000652.1_g760.1.mk promoter
GCTCAAGGCCAAGCCCCACGCCAATGGCCATTGCAAATTCCGCCTTTGTTTCTTGAAAAT
TTGTCAGAAGAAAATACCACAAATTCAGAAGTAAAAACTCTCTACAATTTTAGTATTAGT
ATTGTGTGACTCATAACTGGAAAGCAACTTTGTGTTATCTATCACTTCTAGAAAATACGA
AGACAGAGGGAGAAACTTTAATGCATTTGCTTCTGCAAGCTGAATTGAATTTCTTTTATT
ATTGGAGGCCTAGGGATTTCGAATCTCTGCCCACATTGATGGAGGTGGAATAGCTCAACC
AACTGAGCTATAACCCACTAGTGGTGAATTGAAAATTTGTATAATGGTTGATTATTCAAT
ATTGGTTAAAAGACCCATCCACAAAGCATGTGCACAAGTAAATTTTCAAATAAGGCCTTT
TTTTTAAGGACTTTTTTTATTTAGATAGGCAATAGCATACTAAACTCACACACACTACAC
GGATGTTAAGACTCGAACCAAACAACTACACTAATGGGTTCTTTGCTACACAAGGTCATT
ATTGATTAAACTTCTATGGAGCCAAATAAAGGTTAACATATTTTTACTCATATTGGGCCT
GGGACTGCGAATAATACAGATTATTTTTCCAAATAGGGTTAATGACCTAAATGGTCCCCA
AACTATTGCCATATTATCATTTTGGTCCACCAACTAAAATTTTCAATTCAAACGTCCTTA
AACTTTCAATTCAAACGTCCTTAAACTTTTTATTTTGTACCAAGATGGTCCATCCGTCAA
AGTTTGTGTATTATTCCTTGAAATCGAGGGACAAAATCGTATTTTTATGTGAGTATCCTC
CTCTAAAAGGGTTATTGATCCTAATGGTCCCCCAACTATTGCTCTAGTATCATTTTGGTC
CACCAACTAAAATTTTCATTTGAACCATCCTTCAACATTTTTTTTNNNNNNNNNNNNNNN
NNNNNNNNNNNNNNTATGAATTAAAATAGTAATATATAATTAAAAATTAAAGGAAACTAT
TATAAAACTTAAAAAAATTAAAAAAATTGAATGTCACCAGCCACATAGCTTTTTTTTTTC
GTGAGACTGACCTAACAATTAATAATAAAAAATTCTCTATTGTATAAATTAATTAAAATC
ACATAAATATAAAAATAAAATAACAATTTTTTTAAAAGAGTAAACAATAATTAAAAATTA
AAGGAAACTATTATAGGAACTTGTACCTAATTTTAAAATTGAAAAAATTGAATCTCACCA
CCAACATGGCTTTTTTTTGCTGACATGGACTTAAAAAGTAATAATTTTTTTCTCTATTGT
TTAAATTAATTAAAAATTTTAAAATCAAATAAATACAAAAATAAATAACAATAAAATTGA
CGGACTTTGACGGTAGTACCATCTTGATACAAAAAAAAAAAGAGTTGAAGAACTGTTCAAA
TGAAAATTTTAGTTGGTGGACCAAAATGATACTAGAGCAATAGTTGAGGGACCGGAGGTA
TTTTGGTTTTGAACGATAGAGCTTCTCAAAAAGTCACATATACCCTTCAAAACTCAGTCA
CGTGGCCATCATGTGATCAGAAGTAAAGGAATCTGTAACGGATGGACCATCTTGGTACAA
AATAAAAAGTTTAAGGACGTTTAAATTGAAAATTTTAGTTGGTGACCAAAATGATAATGG
GGCAATAGTTTAGGGACCATTTAGGTCATTAACCCTTTCCAAATATTATGAACTTGCTTG
GGCCAAGGCCCTGCCAGCCAGTAGATGCAAAATATAAAGGAAAGTAGATTCTGAAAATCT
TATCTGCCAATGTCTAAAGACTACAGTCAACAAGCGTGGCATTTGCCTTAGCCTCAAATC
CACCCCACCATGACTTAAAGCTTATTCAATATCAACCTCACAGGTTCTCCAGAACCTCAG
AAAACTGGGACCAGCCATTCAGTTCTCAGTCTACATGTCTTCACTGAATCTACAAAGTTT
GATAATAATAAACCAATTAA
