# Supplementary material for: Cross-talk between transcriptome, phytohormone and HD-ZIP gene family analysis illuminates the molecular mechanism underlying fruitlet abscission in sweet cherry (Prunus avium L)
Source: BMC Plant Biol. 2021 Apr 10;21:173. doi: 10.1186/s12870-021-02940-8 (PMC8035788; doi:10.1186/s12870-021-02940-8)
Supplement: Supplementary file 12 — Additional file 12. The command of differentially expression genes identified by the DESeq 2 R package. [file 12870_2021_2940_MOESM12_ESM.docx]

**The command of differentially expression genes identified by DESeq 2 R package**

library(DESeq2)

database <- CA_vs_CN_count_matrix

database <- as.data.frame(database)

row.names(database) <- database[,1]

database <- database[,-1]

database <- round(as.matrix(database))

condition <- factor(c(rep("CN",3), rep("CA",3)), levels = c("CN", "CA"))

coldata <- data.frame(row.names = colnames(database), condition)

dds <- DESeqDataSetFromMatrix(countData=database, colData=coldata, design=~condition)

dds <- dds[ rowSums(counts(dds)) > 1, ]

dds <- DESeq(dds)

res <- results(dds)

res <- res[order(res$padj),]

diff_gene_all <- subset(res, padj < 0.05 & (log2FoldChange > 1 | log2FoldChange < -1))

diff_gene_list <- row.names(diff_gene_all)

resdata <- merge(as.data.frame(res), as.data.frame(counts(dds)),by="row.names",sort=TRUE)

write.csv(resdata,file = "CA_CN_allgene.csv",row.names = F)

write.csv(diff_gene_list,file = "CA_CN_DEGlist.csv")

write.csv(diff_gene_all,file = "CA_CN_DEG.csv")
